# Optimization of phenolics recovery from *strychnos nux-vomica* L. seed applying deep eutectic solvent-ultrasound assisted extraction

**DOI:** 10.1038/s41598-026-47748-4

**Published:** 2026-04-17

**Authors:** Haroon Iftikhar, Sumia Akram, Noor Ul Ain Khalid, Shan Ahmed Khan, Aqsa Batool, Dildar Ahmed, Muhammad Mushtaq

**Affiliations:** 1 Department of Chemistry, Institute of Chemical Sciences, Government College University, Lahore, 54000 Pakistan; 2 Division of Science and Technology, University of Education, Lahore, 54770 Pakistan; 3 Department of Chemistry, Forman Christian College (A Chartered University), Lahore, 54600 Pakistan; 4 Department of Chemistry, Pakistan Institute of Engineering and Applied Sciences, Islamabad, 45650 Pakistan; 5 Department of Energy and Petroleum Engineering, University of Stavanger, Stavanger, Norway

**Keywords:** *Strychnos nux-vomica* L. seeds, DES-Ultrasound extraction, RSM-ANN optimization, SEM-EDX, Chemistry, Plant sciences

## Abstract

*Strychnos nux-vomica* L. seeds (SNV) are a rich source of bioactive compounds with antioxidant and antidiabetic properties. In this study, the deep eutectic solvent-ultrasound assisted extraction (DES-UAE) is applied for the extraction of bioactive compounds from SNV seeds, which significantly enhances extraction efficiency, while reducing environmental impact. DES made up of ammonium acetate-propanoic acid (1:3) in combination with ethanol and UAE offered higher recovery rates of SNV bioactive. The extraction process was optimized using response surface methodology (RSM) along with artificial neural networking (ANN), considering extraction time (X_1_), temperature (X_2_), and DES/feed ratio (X_3_) as the independent variables. The dependent variables (responses) evaluated were extraction yield, the total phenolic and flavonoid (TP and TF) content, antioxidant capacity (AC), and antidiabetic (AD) potential. Optimal extraction conditions (25 min, 40 °C temperature, 30 mL/g DES/feed ratio) at 40 kHz ultrasound frequency resulted in 6.31% yield, 64.72 (mg GAE/g) dw TP, 103.19 mg (QE/g) dw TF, 89.87% AC, and 68.53% antidiabetic (AD) potential. The model’s reliability was confirmed through smaller (≤ 5%) relative standard deviation (RSD), root mean square error (RMSE), percentage prediction error (PPE), absolute average deviation (AAD), and R² values. Scanning electron microscopy-energy dispersive X-ray spectroscopy (SEM-EDX) analysis showed effective cell disruption, supporting enhanced extraction. The outcomes of the present research recommend that DES-based solvent in combination with UAE can be further explored for sustainable and efficient extraction of phenolic bioactives.

**Supplementary Information:**

The online version contains supplementary material available at 10.1038/s41598-026-47748-4.

## Introduction

Plants are known as nature’s pharmacies for the presence of many beneficial phytochemicals that either alone or in combination with others act as natural remedies against diseases. According to the world health organization (WHO), about 80% of the population around the globe relies on natural product-based medicines^1^. Interestingly, 25% of the total modern pharmacopoeia still uses plant-based extracted drugs^2^. *Strychnos nux vomica* L. (SNV) is an evergreen plant known for its uses in folk medicines^3^. This tree generally attains a maximum height of 10–25 m and occurs in Sri Lanka, Australia, and some parts of Asia. SNV seeds flower during March-April and begin to fruit May-November. The seeds of the SNV are very hard and disk-shaped with a concave structure. The SNV seeds typically grow 2–3 cm in diameter, 3–7 mm thick, and hispid on their surface^4^.

SNV seeds carry a long use history in folk medicine to treat numerous digestive disorders^5^ and degenerative health conditions, such as oxidative stress and diabetes^6^. Natural compounds found in these SNV seeds include terpenoids, flavonoids, polyphenols, sterols, tannins, saponins, and lipids^7^. The raw seeds are toxic due to strychnine and brucine, which can cause severe muscle spasms and respiratory failure^6^. Therefore, the seeds must be prepared carefully to make them safe for therapeutic use^8^. SNV seeds contain phenolics which are particularly excellent at fighting oxidative stress and diseases associated with it ^9,10^. Type II diabetes (DB-II), like many others is most dominant stress-initiated health disorder which involves insufficient production of insulin or its ineffective use. A set of synthetic drugs are available in the market to treat DB-II but majority of these cause after effects. Plant secondary metabolites particularly phenolics have been shown to act as a blood sugar regulator either via inhibitors of alpha-amylase or insulin modulators^11^.

Response surface methodology (RSM) has become a fascinating tool to study and optimize the complex processes involving interaction between the input variables. RSM builds approximation vector of true response as function of input variables and then follow this vector to locate the area of highest steepness (maxima/minima) of response. Artificial neural networking (ANN), on the flip side try to develop the nature of correlation between the input variables and response like biological neural networks. Under such a layer structure, input variables go through intermediate layers, and finally, output values and their networking can accurately approximate complex relationships in data. Using ANN for non-linear models provides a strong modeling approach, which mimics the neural processes of the human brain. This advanced technique has served as a reliable means of dealing with the optimization problems of diverse disciplines in science^12^.

Developing an efficient, benign, and sustainable extraction strategy has been a challenging task to benefit from nature’s pharmacies. A cascade extraction solvents and techniques have been proposed but majority of these were ruled out either for their limited recovery rates, detrimental effects, or environmental issues. For example, supercritical fluid-based extraction (SFE) stands green and efficient but could not be universalized for higher capital investment^13^. Likewise, enzyme assisted extraction involve the unrevealed hydrolysis products besides the use of sensitive and high cost reagents^14^. Microwave-assisted extraction techniques, likewise other thermal heating methods, can degrade the bioactive compounds; however, the ultrasound technique can accelerate mass transfers and breakdown of the cell walls through cavitation bubble^15^, but it still requires an efficient and green extraction solvent. Recently, deep eutectic solvents (DES) have emerged as non-volatile, tunable, and benign solvents that are widely explored for green synthesis, extraction, and biomass conversion^16^. DES can be prepared by combining hydrogen bond donor and acceptor compounds in different stoichiometric ratio^17^. Albeit, DES are being declared benign and non-volatile extraction solvent, their higher viscosities limit their application as an extraction solvent.

Keeping in view the therapeutic potential of SNV, we have applied a deep eutectic liquid made up of ammonium acetate (AA) and propanoic acid (PA) with 1:3 (AA/PA) in combination with ultrasound to extract *Strychnos nux-vomica* L. seed phenolics. The extraction conditions involving extraction conditions including time, temperature, solvent/feed ratio, ultrasound time and power were tested and optimized for the enhanced recovery of phenolics. For the quality check, the extracts obtained under various conditions were tested for their total phenolic (TP), total flavonoid (TF), antioxidant capacity (AC), and antidiabetic (AD) potential. Finally, the observed values of TP, TF, AC, and AD were subjected to artificial neural networking (ANN) to establish the correlation between the parameters tested and observed responses. The outcomes of this study may introduce new opportunities regarding DES-based green and sustainable extraction of plant bioactives.

## Materials and methods

### Chemicals and reagents

The chemicals, reagents, and solvents used during the present research were of known purity, manufacturing date, and source. Ammonium acetate (≥ 95%), propanoic acid (≥ 98%), sodium sulfite (≥ 90.0%), alpha-amylase (≥ 97.0%), biuret reagent, and (≥ 95.0%) DPPH were purchased from Merk (Darmstadt, Germany). Folin-Ciocalteu reagent (2*N*) were obtained from Scharlau Chemicals S.A., Barcelona, Spain. Acarbose, 3,5-dinitrosalicylic acid (≥ 97%), starch (≥ 90%), and quercetin (≥ 99%) were supplied by Sigma Aldrich (St Louis, USA).

### Purchase and preparation of SNV

*Strychnos nux-vomica* L. (SNV) seeds were purchased from the local herbal market in Lahore (31°34’44.9"N 74°19’22.6"E), Pakistan and identified by Prof. Dr Tehreema Iftikhar (plant taxonomist). The specimen was stored in Dr Sultan Ahmad Herbarium of Government College University, Lahore, Pakistan) under specimen voucher number 3081 and can be accessed from https://gcu.edu.pk/sultanherbarium.php. The seeds were then washed to remove dust, shade-dried, and ground using AG-639 grinder into coarse powder of uniform particle size and passed through 80 mesh sieves for uniform particle size, critical for consistent extraction efficiency in subsequent experiments.

### Preparation of DES

The ammonium acetate and propanoic acid (AA-PA) DES was prepared using a 1:3 mol ratio, respectively. The accurately measured 385 g (5 moles) of AA and 1078 g (15 moles) of PA were taken into a flask connected to a condenser and tube having silica gel to remove moisture. The mixture was continuously stirred under vacuum at temperature range of 60–70 °C for seven hours. This procedure resulted in the formation of a clear, colorless liquid, identified as AA-PA DES, which was subsequently subjected to spectroscopic and physicochemical analysis^17^.

### DES-Ultrasound assisted extraction

For the extraction process, 1.0 gram of *Strychnos nux-vomica* (SNV) seed powder was mixed with varying volumes (10–30 mL) of DES, following the experimental design outlined in Tables 1 and 2. The prepared mixtures were then exposed to ultrasonic treatment using a 2.0 L bath-type ultrasound bath having a frequency of 40 kHz (Model SS-Z120, Anakonia, Guangdong, China). The duration of sonication varied according to the time intervals specified in Table 1. The amount of extract obtained (after filtration) was compared with the weight of pure DES. For calculating the % yield, the weight of the pure DES is subtracted from the weight of the extract with DES and divided by the dry weight of the seed (1 g). Equation 1 gives formula^18^.

1 $$\:\mathbf{\%}\:\mathbf{Y}\mathbf{i}\mathbf{e}\mathbf{l}\mathbf{d}\:=\frac{\mathbf{W}\mathbf{e}\mathbf{i}\mathbf{g}\mathbf{h}\mathbf{t}\:\mathbf{o}\mathbf{f}\:\mathbf{e}\mathbf{x}\mathbf{t}\mathbf{r}\mathbf{a}\mathbf{c}\mathbf{t}\:\mathbf{w}\mathbf{i}\mathbf{t}\mathbf{h}\:\mathbf{D}\mathbf{E}\mathbf{S}\:-\:\mathbf{P}\mathbf{u}\mathbf{r}\mathbf{e}\:\mathbf{D}\mathbf{E}\mathbf{S}\:\mathbf{w}\mathbf{e}\mathbf{i}\mathbf{g}\mathbf{h}\mathbf{t}}{\mathbf{D}\mathbf{r}\mathbf{y}\:\mathbf{p}\mathbf{o}\mathbf{w}\mathbf{d}\mathbf{e}\mathbf{r}\:\mathbf{w}\mathbf{e}\mathbf{i}\mathbf{g}\mathbf{h}\mathbf{t}\:\mathbf{o}\mathbf{f}\:\mathbf{s}\mathbf{e}\mathbf{e}\mathbf{d}}\times\:100$$

### SEM-EDX analysis

The surface morphology of *Strychnos nux-vomica* seeds before and after the extraction was photographed using Scanning Electron Microscope (Zeiss model no EVO LS10) at a voltage of 20.0 kV (C2DX signal). SEM was coupled with energy-dispersion X-ray (EDX) to detect the variation in elemental percentage of SNV seed before and after extraction.

### Optimization of extraction conditions

Based on initial trials and previous studies by our research group^19^, three extraction parameters, i.e. ultrasound treatment time (X_1_), temperature (X_2_) and DES/feed (X_3_) were investigated over three levels (-Ҡ, 0, +Ҡ) following the central composite design (CCD) (Table 1). The CCD is thought to be a robust and economical experimental layout to investigate the numerical factors affecting multiple responses^20^. A set of seventeen experiments comprising five replicates at the center point (0) and twelve at the factorial points (±Ҡ) were conducted to calculate extract Yield, TP, TF, AC, and AD, respectively.

**Table 1:** Experimental factor levels employed for extracting compounds from SNV seeds using DES.

Independent variables	Codes	Units	Levels		
			-Ҡ	0	+Ҡ
Ultrasound Time	X_1_	Minutes (min)	10	25	40
Extraction Temperature	X_2_	Degree Celsius (ºC)	40	45	50
Solvent/feed ratio	X_3_	Milliliters per gram (mL/g)	10	20	30

The responses obtained under the above-mentioned conditions were subjected to statistical evaluation using Design Expert 13 software and factors affecting the response significantly (*p* ≤ 0.05) were modulated following Eq. 2.

2 $$\:\boldsymbol{y}={\boldsymbol{\beta\:}}_{\boldsymbol{o}}+\sum\:_{\boldsymbol{i}=1}^{\boldsymbol{k}}{\boldsymbol{\beta\:}}_{\boldsymbol{i}}{\boldsymbol{X}}_{\boldsymbol{i}}+\sum\:_{\boldsymbol{i}=1}^{\boldsymbol{k}}{\boldsymbol{\beta\:}}_{\boldsymbol{i}\boldsymbol{i}}{\boldsymbol{X}}_{\boldsymbol{i}}^{2}+{\boldsymbol{\Sigma\:}}_{\mathbf{i}=1}^{\mathbf{k}}\:{\sum\:_{\boldsymbol{j}=\boldsymbol{i}+1}^{\boldsymbol{k}-1}{\boldsymbol{\beta\:}}_{\boldsymbol{i}\boldsymbol{j}}\:\boldsymbol{X}}_{\boldsymbol{i}}{\boldsymbol{X}}_{\boldsymbol{j}}+\:\epsilon$$

In Eq. 2, $\:\boldsymbol{y}$ represents the observed responses while X_i_ to X_j_ indicate the independent variables. The term $\:{\boldsymbol{\beta\:}}_{\boldsymbol{i}}$ corresponds to the regression coefficients, k denotes the levels used in the design, and $\epsilon$  refers to the random or experimental error 

### Optimization using artificial neural networking (ANN)

ANN was employed to capture the non-linear relationships between (X₁), (X₂), and (X₃) and the response parameters, which included bioactive yield (%), TP and TF, antioxidant (AC), and antidiabetic (AD) activity. The real time observed values against the conditions stated in Table 2 were further processed in ANN. A feedforward backpropagation neural network was constructed and evaluated using R², RMSE, and MSE metrics. The *Levenberg-Marquardt* (LM) algorithm was selected as the training function, with the mean square error (MSE) distributed among the validation, testing, and training datasets at 15, 15, and 70%, respectively. Weight and bias adjustments were carried out using the gradient descent method available in MATLAB’s Neural Network Toolbox (*nntools*). The ANN structure included three hidden layers, configured with three input neurons (X₁, X₂, X₃), followed by hidden layers using 20 neurons with a Logsig transfer function, 10 neurons with a Tansig function, and a final output layer using Purelin (linear activation function). ANN optimization was executed using MATLAB 2019a.^21^.

### Estimation of total phenolic content assay

Total phenolic content (TP) in the extracts of SNV produced by DES-UAE was estimated using Folin Ciocalteu reagent (FCR) as reported by (Ainsworth et al., 2007) with some modifications^22^. In this method, 1.0 mL of SNV seed extract (sample), Gallic acid standard (positive control), and pure DES (blank) were separately mixed with 0.3 mL of 2*N* FCR, and then 4.5 mL of distilled water was added. After that, the whole mixture was kept in the dark for 8 min at 25 ℃. After the completion of incubation, 10 mL of 7.4% Na_2_CO_3_ was mixed into the mixture and then kept in the dark for 30 min at 40 ℃. The sample containing the extraction solvent only was used in blank preparation. The absorbance of the reaction mixture was observed at 765 nm.

### Estimation of total flavonoid (TF) content assay

To determine the TF content present in SNV seeds, 0.3 mL of SNV seeds extract obtained via DES-UAE was diluted with 3.4 mL of methanol-water solution (30% (v/v)), which was further added with 1.5 µL of 0.5 M NaNO_2_ solution and 1.5 µL of 0.3 M aluminum chloride solution. After 5 min, 1 mL of sodium hydroxide (1 M) was added to this solution. The absorbance of the mixture was observed at 565 nm using a spectrophotometer. For the preparation of the blank, the SNV sample was replaced with pure DES. Quercetin was utilized as a calibration standard^23^.

### Antidiabetic activity assay (AD)

The in vitro antidiabetic potential of SNV seed extracts produced by DES-UAE was monitored in terms of their alpha-amylase inhibitory activity. In this assay, 1 mL of alpha-amylase enzyme solution dissolved in buffer (0.5 international units per milliliter) was mixed with an equal volume of SNV extract (1.0 mg/mL) and further diluted with 5 mL of pH 7.0 phosphate buffer. After an incubation of 30 min, 1.0 mL of starch solution was added to each sample, and the solutions were incubated for another 3 min. Now, 1.0 mL of 0.8 M DNS was added after the incubation, and each sample was placed in hot water for 15 min at 85 ℃. Finally, 10 mL of distilled water was added to the reaction mixture, and the absorbance was measured at 545 nm using a spectrophotometer^24^. Equation 3 was used to calculate AD activity (%).

3 $$\:\mathrm{\%}\:\mathrm{A}\mathrm{l}\mathrm{p}\mathrm{h}\mathrm{a}-\mathrm{a}\mathrm{m}\mathrm{y}\mathrm{l}\mathrm{a}\mathrm{s}\mathrm{e}\:\mathrm{i}\mathrm{n}\mathrm{h}\mathrm{i}\mathrm{b}\mathrm{i}\mathrm{t}\mathrm{o}\mathrm{r}\mathrm{y}\:\mathrm{a}\mathrm{c}\mathrm{t}\mathrm{i}\mathrm{v}\mathrm{i}\mathrm{t}\mathrm{y}\:=\frac{\:{\boldsymbol{A}\boldsymbol{b}\boldsymbol{s}}_{\boldsymbol{c}\boldsymbol{o}\boldsymbol{n}\boldsymbol{t}\boldsymbol{r}\boldsymbol{o}\boldsymbol{l}}\:-{\boldsymbol{A}\boldsymbol{b}\boldsymbol{s}}_{\boldsymbol{s}\boldsymbol{a}\boldsymbol{m}\boldsymbol{p}\boldsymbol{l}\boldsymbol{e}}\:}{{\boldsymbol{A}\boldsymbol{b}\boldsymbol{s}}_{\boldsymbol{c}\boldsymbol{o}\boldsymbol{n}\boldsymbol{t}\boldsymbol{r}\boldsymbol{o}\boldsymbol{l}}\:}\times\:100$$

### Antioxidant activity assay

The antioxidant capacity was assessed in terms of DPPH radical scavenging capacity (DPPH-RSC). The extracts of SNV seed obtained by DES-UAE were treated with DPPH free radicals following the protocol documented by Lin, et al. ^25^ with slight modifications. In short, a 1.5 ppm solution of DPPH in methanol and further diluted with methanol until absorbance reached to 0.99 at 517 nm. Now, 1.0 ml of SNV seed extracts prepared in DES-ethanol mixture (1:1) was mixed with 4 mL of DPPH solution under dark conditions and incubated for another 30 min at 25 ℃. Finally, absorbance was measured using a spectrophotometer at 520 nm. Equation 4 was used to calculate DPPH-RSC.

4 $$\:\mathrm{D}\mathrm{P}\mathrm{P}\mathrm{H}-\mathrm{R}\mathrm{S}\mathrm{C}\:\left(\mathrm{\%}\right)=\frac{\:{\boldsymbol{A}\boldsymbol{b}\boldsymbol{s}}_{\boldsymbol{c}\boldsymbol{o}\boldsymbol{n}\boldsymbol{t}\boldsymbol{r}\boldsymbol{o}\boldsymbol{l}}\:-{\boldsymbol{A}\boldsymbol{b}\boldsymbol{s}}_{\boldsymbol{s}\boldsymbol{a}\boldsymbol{m}\boldsymbol{p}\boldsymbol{l}\boldsymbol{e}}\:}{{\boldsymbol{A}\boldsymbol{b}\boldsymbol{s}}_{\boldsymbol{c}\boldsymbol{o}\boldsymbol{n}\boldsymbol{t}\boldsymbol{r}\boldsymbol{o}\boldsymbol{l}}\:}\times\:100$$

### Statistical analysis

Different statistical tests, including percentage related standard deviation (% RSD), percentage prediction error (PPE), RMSE (root mean square error), regression coefficient (R^2^), and AAD (absolute average deviation), were used as a tool for interpreting the comparison of performance between ANN and RSM-generated models. SPSS was used for t-test analysis to find the statistical differences between ANN and RSM-generated models.

5 $$\:\mathrm{\%}\:\mathrm{R}\mathrm{S}\mathrm{D}\:=\:\frac{Standard\:Deviation\:}{Mean\:}\times\:100$$

6 $$\:Percentage\:P\mathrm{r}\mathrm{e}\mathrm{d}\mathrm{i}\mathrm{c}\mathrm{t}\mathrm{i}\mathrm{o}\mathrm{n}\:\mathrm{e}\mathrm{r}\mathrm{r}\mathrm{o}\mathrm{r}\:=\:\frac{Predicted\:Value-Measured\:VAlue}{Predicted\:value}*100$$

$$\:RMSE=\sqrt{\frac{1}{n}\sum\:_{i=1}^{n}{\left({y}_{i}-{Y}_{i}\right)}^{2}}$$

$$\:{R}^{2}=1-\frac{\sum\:_{i=1}^{n}{\left({y}_{i}-{Y}_{i}\right)}^{2}}{\sum\:_{i=1}^{n}{\left({Y}_{i}-\stackrel{-}{y}\right)}^{2}}$$

$$\:AAD=\left[\frac{\sum\:_{1=1}^{n}\left|{Y}_{i}-{y}_{i}\right|/{Y}_{i}}{n}\right]\times\:100$$

## Results and discussions

Table 2 shows the actual results of DES-UAE extraction carried out from the SNV seed under different conditions, along with RSM and ANN predicted data. The extraction yield ranged from 0.40 to 7.10%, with (Run 12) achieving the highest yield at 40 min and 45 °C using 10 mL/g solvent, while the lowest yield was observed at (Run 10) (10 min, 50 ℃, and (DES)/feed ratio (20 mL/g)). From the findings, it is concluded that the prolonged sonication time at optimal temperature can offer better extraction efficiencies as previously noted by Dhanani, et al. ^26^ who investigated phytochemical extraction from *Withania somnifera*. It should also be mentioned here that the effect of temperature was found to be more complicated; perhaps it shows a quadratic effect on overall percentage yield as observed by Kaleem, et al. ^27^. The ultrasound treatment was found to enhance the extraction efficiency significantly by promoting cell rupturing and helping in the quicker release of phytochemicals into the solvent^19^. Dheyab et al., (2021) reviewed that DESs are efficient green medium for the extraction of phytochemicals^28^. L-carnitine and 1,3 butanediol-based DES coupled with UAE for the extraction of gingerols showed high recovery of 3.82 mg/g ^29^.

The range of total phenolic content (TP) spanned from 28.60 mg GAE/g (Run 11) (X_1_ = 40, X_2_ = 45 and X_3_ =10), to 85.23 mg GAE/g (Run 2), showing that the extraction conditions of (X_1_ = 40, X_2_ = 45 and X_3_ = 30) solvent produced the maximum phenolic content. This indicates that the incubation time, temperature, and DES/feed ratio affected the total phenolic content in SNV produced by DES-UAE. A similar kind of behavior for orbital type shaking has already been observed^30^. The maximum phenolic content obtained from SNV during this research using DES-UAE, was 85.23 mg GAE/g, which is higher than that found in SNV’s methanolic extract, (71 mg GAE/g), which was achieved after performing extraction three times, requiring approximately three days^31^. This finding proves that AA/PA DES is a better and sustainable alternative to conventional solvents. The same type of study is also reported by Illoussamen et al., (2025) in which choline chloride-based DES demonstrated superior phenolics recovery from *alperujo* using ultrasound-assisted extraction coupled with DES in comparison to conventional solvents. The DES-UAE phenolic extraction from *alperujo* was 585.90 mg GAE/g, which is four times better than water (114.53 mg GAE/g) and almost twelve times better than ethanol (16.09 mg GAE/g) ^32^.

**Table 2:** A comparison of true values versus results with RSM and ANN-predicted values for extraction yield (%), TP and TF, antioxidant (AC), and antidiabetic (AD) activities *X₁*,* extraction time (min); X₂*,* extraction temperature (°C); X₃*,* solvent-to-feed ratio (mL/g); TP*,* total phenolic content; TF*,* total flavonoid content.*,

NO of Runs	X_1_	X_2_	X_3_	% Yield			TP			TF			Antioxidant activity (AC)			Antidiabetic activity (AD)		
	Min	ºC	mL/g	Actual-Exp	RSM-Predicted	ANN-Predicted	Actual-Exp	RSM-Predicted	ANN-Predicted	Actual-Exp	RSM-Predicted	ANN-Predicted	Actual-Exp	RSM-Predicted	ANN-Predicted	Actual-Exp	RSM-Predicted	ANN-Predicted
**1**	40	50	20	3.5	3.15	3.5	71.83	68.32	71.83	124.77	126.4	124.73	90.11	89.88	89.82	69.01	68.74	69.08
**2**	40	45	30	5.1	5.2	5.1	85.23	86.85	85.23	131.43	126.06	131.59	89.36	89.49	89.36	74.65	75.27	74.65
**3**	10	45	10	4.6	4.5	4.6	71.79	70.16	68.95	49.99	55.35	54.42	89.81	89.68	89.69	71.54	70.92	71.54
**4**	40	40	20	4.3	4.25	4.3	51.72	52.03	51.72	140.82	139.98	140.82	90.03	89.87	90.03	67.61	66.82	67.61
**5**	25	45	20	4.8	5.1	5.05	69.47	71.22	71.45	130.5	128.16	128.67	83.66	83.93	83.86	69.22	68.86	68.74
**6**	25	45	20	5.1	5.1	5.05	69.52	71.22	71.45	124.2	128.16	128.67	85.13	83.93	83.86	68.87	68.86	68.74
**7**	10	40	20	3.9	4.25	3.9	69.29	72.81	69.29	141.78	140.14	141.76	90.03	90.26	89.85	65.16	65.43	65.16
**8**	10	45	30	1.3	1	1.18	63.82	62.23	66.05	128.54	123.97	128.52	90.18	89.92	90.18	79.34	78.9	79.2
**9**	25	45	20	5.5	5.1	5.05	70.16	71.22	71.45	128.01	128.16	128.67	85.11	83.93	83.86	67.46	68.86	68.74
**10**	10	50	20	0.4	0.45	0.4	63.22	62.91	65.53	99.78	100.62	99.75	87.24	87.4	87.24	74.01	74.8	74.01
**11**	40	45	10	2.7	3	2.65	28.6	30.18	29.32	74.31	78.88	71.42	91.94	92.2	91.84	69.44	69.88	69.44
**12**	25	40	10	7.1	6.85	7.1	32.09	30.19	32.09	54.61	50.89	54.61	87.77	87.67	88.27	69.81	70.16	69.81
**13**	25	50	10	4.4	4.45	4.4	40.13	42.06	40.13	31.82	25.61	31.82	90.11	90.09	90.11	67.93	67.76	67.93
**14**	25	45	20	5.1	5.1	5.05	71.62	71.22	71.45	132.29	128.16	128.67	81.52	83.93	83.86	68.21	68.86	68.74
**15**	25	40	30	6.3	6.25	5.77	65.16	63.23	65.16	103.86	110.06	104.51	90.26	90.29	90.26	68.63	68.8	68.65
**16**	25	45	20	5	5.1	5.05	75.35	71.22	71.45	125.8	128.16	128.67	84.24	83.93	83.86	70.56	68.86	68.74
**17**	25	50	30	3.5	3.75	3.5	55.85	57.75	55.85	78.51	82.24	78.5	84.92	85.02	84.92	82.86	82.5	83.14

The highest flavonoid content (TF) value reached 141.78 mg QE/g at (Run 7) (X_1_ = 10, X_2_ = 40 ℃ and X_3_ = 20). The minimum TF value obtained was 31.82 mg QE/g (Run 13) at a time of 25 min, 50 ℃, and a DES/feed ratio of 10 mL/g. It is evident from the observed data that shorter sonication intervals resulted in higher TF and vice versa. A similar type of finding for methanolic solvent-based extraction of flavonoids from SNV has been reported by Lin, et al. ^33^. It can be speculated that prolonged sonication after optimal value can degrade the flavonoids through free radical oxidation, thermal stress, and shear forces. Contrarily, an increase in temperature enhanced the TF at 50 °C, but increasing the temperature further will negatively affect the TFC and cause a decrease in the TFC of SNV extracts obtained by DES UAE. This might be because at elevated temperature, cavitation bubbles that are produced by sonication can generate free radicals^34^, while a higher solvent-to-solid ratio helps in better flushing of flavonoids^35^. The lower recovery of total flavonoid content (TF) compared to conventional solvents in polar DES-UAE extraction is previously reported by Vo et al., (2023) from *Garcinia mangostana* L. The reason for the lower recovery of flavonoids is the presence of several methoxyl groups, making them less polar than phenolic compounds^36^.

The minimum value of antioxidant activity (AC) measured in terms of DPPH-RSC was 83.66% for SNV extract obtained under DES-UAE of Run 5 (Table 2). The maximum antioxidant value obtained was 91.94% at (X_1_ = 40, X_2_ = 45 and X_3_ = 10) (Run 11). For antioxidant activity, extracts obtained at lower DES/feed ratios (mL/g) showed higher percentage inhibition of DPPH radicals. This might have happened due to the fact that with less solvent to solid ratio, the extract is more concentrated, so in testing a fixed volume, more antioxidant molecules per unit volume are present that lead to higher percentage inhibition. The same type of effect was previously reported by Isnindar et al., (2025) on *Premna serratifolia* L. leaves in which 1:10 g/mL ratio showed the higher antioxidant activity (IC_50_ = 11.99 ± 1.02 µg/mL) as compare to 1:20 g/mL and 1:30 g/mL ^37^. Martinović et al., (2022) reported that the antioxidant (DPPH radical scavenging) potential of green tea leaves was significantly improved by using citric acid-sorbitol-based DES compared to water and ethanol. The DES extract showed a better DPPH radical scavenging activity with IC_50_ of 0.44 mg/mL in comparison to water (0.42 mg/mL) and ethanol (0.24 mg/mL)^38^. These findings prove that AA/PA (polar DES) is a better alternative to conventional solvents.

The antidiabetic (AD) activity of SNV exhibited a broad range, with the lowest value of 65.16% (Run 7) at 10 min, 40 °C, and 20 mL/g and the highest value 82.86% (Run 17), where the superior antidiabetic potential at the time of 25 min, 50 ℃ temperature, and DES/feed ratio of 30 mL/g correlates with enhanced extraction of bioactive compounds. This is because more extraction time at high temperature with an appropriate (optimal) DES/feed ratio helps in better extraction of phytochemicals that are responsible for antidiabetic activity. Zengin et al., (2022) reported that the DES-extract of *Cytinus hypocistis* markedly showed higher antidiabetic potential (up to 2.54 mmol ACAE/g) compared to conventional solvents^39^.

### Model equations and optimization using RSM

Table 3 compares the coefficients of linear, interaction, and quadratic coefficient for mathematical modulation of yield (%), TP, TF, AC, and AD following Eq. 1. A positive value of co-efficient indicates that an increase in input increases the response and vice versa. The ANOVA and fit statistics analysis of applied experimental design have been assembled in Table 4. The probability *p ≤* 0.05 *was* followed to significance level, in this context, the model *p* values for yield (%), TP, TF, AC, and AD were < 0.01 establishing that the selected extraction conditions play significant role with high R^2^ (> 0.90) and low % CV, therefore indicating very strong model fitness and precision. All responses follow second order regression model with most prominent quadratic effect in TF and antidiabetic (AD) activity. The precision of antioxidant and antidiabetic models is excellent, whereas the precision of yield is low with higher variability (CV = 8.11%) and less predictive accuracy (R^2^ = 0.7810). The experimental design followed to investigate the DES-UAE based extraction and subsequent activities can do good prediction of responses.

**Table 3:** Coefficient estimates for %yield, TP and TF, antioxidant and antidiabetic activity to modulate Eq. 2.

Factors	% Yield	TP	TF	Antioxidant (AC)	Antidiabetic (AD)
**Intercept**	5.10	71.22	128.16	83.93	68.86
**X**_**1**_	0.6750	-3.84	6.40	0.5225	-1.17
**X**_**2**_	-1.22	1.60	-13.28	-0.7138	2.83
**X**_**3**_	-0.3250	12.18	28.95	-0.6137	3.34
**X**_**1**_ **X**_**2**_	0.6750	6.55	6.49	0.7175	-1.86
**X**_**1**_ **X**_**3**_	1.43	16.15	-5.36	-0.7375	-0.6475
**X**_**2**_ **X**_**3**_	-0.0250	-4.34	-0.6378	-1.92	4.03
**X**_**1**_^**2**^	-1.99	3.42	13.75	3.74	0.7593
**X**_**2**_^**2**^	-0.0875	-10.63	-15.12	1.68	-0.6757
**X**_**3**_^**2**^	0.3125	-12.29	-45.84	2.65	4.12

*X₁*,* extraction time (min); X₂*,* extraction temperature (°C); X₃*,* solvent-to-feed ratio (mL/g); TP*,* total phenolic content; TF*,* total flavonoid content.*

Finally, the lack fit F-values for % yield, TP, and TF, antioxidant (AC), and antidiabetic (AD) activity calculated to be 2.97, 2.74, 6.41, 0.0522, and 0.7076, respectively, indicates that lack of fit is not significant which implies the experimental design followed possess good fit.

**Table 4:** Analysis of Variance (ANOVA) presented in terms of mean sum of squares for comparing the effectiveness of tested variable for % yield, TP and TF, antioxidant and antidiabetic activity.

Factors	ANOVA for the quadratic model													RSM			%CV
	Mean sum of squares									Mean Sum of Squares of model	Mean sum Square For error	*p*-value of model	*p*-value lack of fit				
	Linear terms			Linear combined terms			Quadratic terms										
	**X**_**1**_	**X**_**2**_	**X**_**3**_	**X**_**1**_ **X**_**2**_	**X**_**1**_ **X**_**3**_	**X**_**2**_ **X**_**3**_	**X**_**1**_^**2**^	**X**_**2**_^**2**^	**X**_**3**_^**2**^					**R** ^**2**^ **-Values**			
														**R** ^**2**^	**Adjusted R** ^**2**^	**Predicted R** ^**2**^	
**Yield (%)**	3.65	12.00	0.8450	1.82	8.12	0.0025	16.63	0.0322	0.4112	43.40	0.12	<0.0001*	0.1599	0.9810	0.9566	0.7810	8.11
**TP**	118.09	20.40	1187.44	171.45	1043.05	75.21	49.30	475.70	635.70	3807.74	10.59	<0.0001*	0.1775	0.9809	0.9563	0.7847	5.25
**TF**	328.14	1409.85	6704.92	168.26	114.78	1.63	795.69	962.65	8848.33	19328.46	36.37	<0.0001*	0.0523	0.9870	0.9703	0.8243	5.69
**Antioxidant (AC)**	2.18	4.08	3.01	2.06	2.18	14.75	58.86	11.91	29.60	138.94	1.31	0.0018	0.9821	0.9382	0.8587	0.8698	1.30
**Antidiabetic (AD)**	10.90	63.85	89.51	13.88	1.68	64.88	2.43	1.92	71.45	320.86	1.18	<0.0001*	0.5958	0.9749	0.9426	0.8350	1.53

*X₁*,* extraction time (min); X₂*,* extraction temperature (°C); X₃*,* solvent-to-feed ratio (mL/g); TP*,* total phenolic content; TF*,* total flavonoid content.*, * The probability (*p*)-values ≤ 0.001 indicates that the model terms are significant at 99% confidence level.

**Fig. 1:**
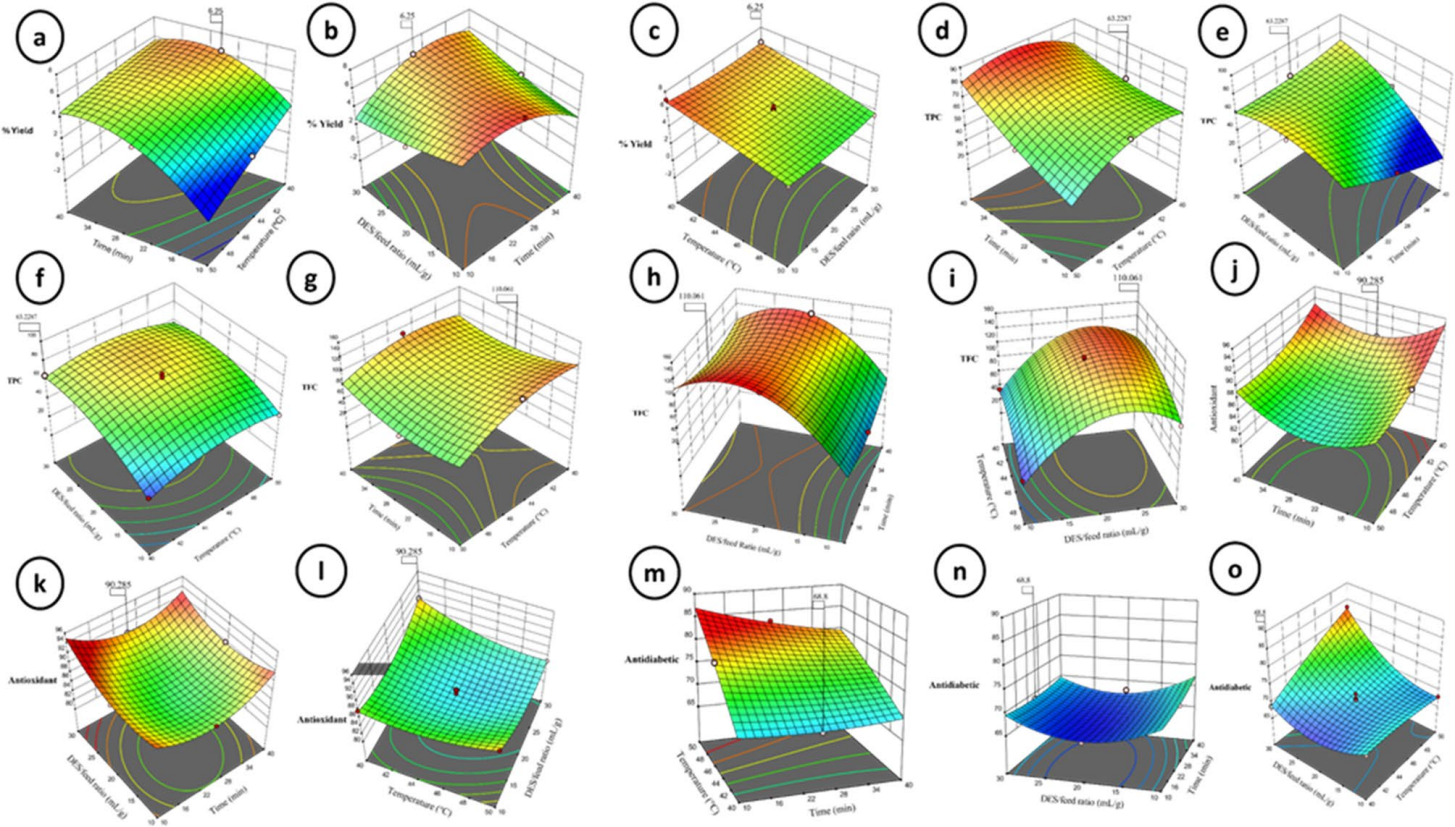
Images from (a) to (o) show how time, temperature, and DES/feed ratio effects %yield, TP and TF, antioxidant (AC) and antidiabetic (AD) activity. Time and temperature show the quadratic effect on % yield, TP and AD, while TF is mostly influenced by DES/feed ratio. High solvent ratio and increased the antioxidant activity (AD) up to optimal value of temperature.

**Fig. 2:**
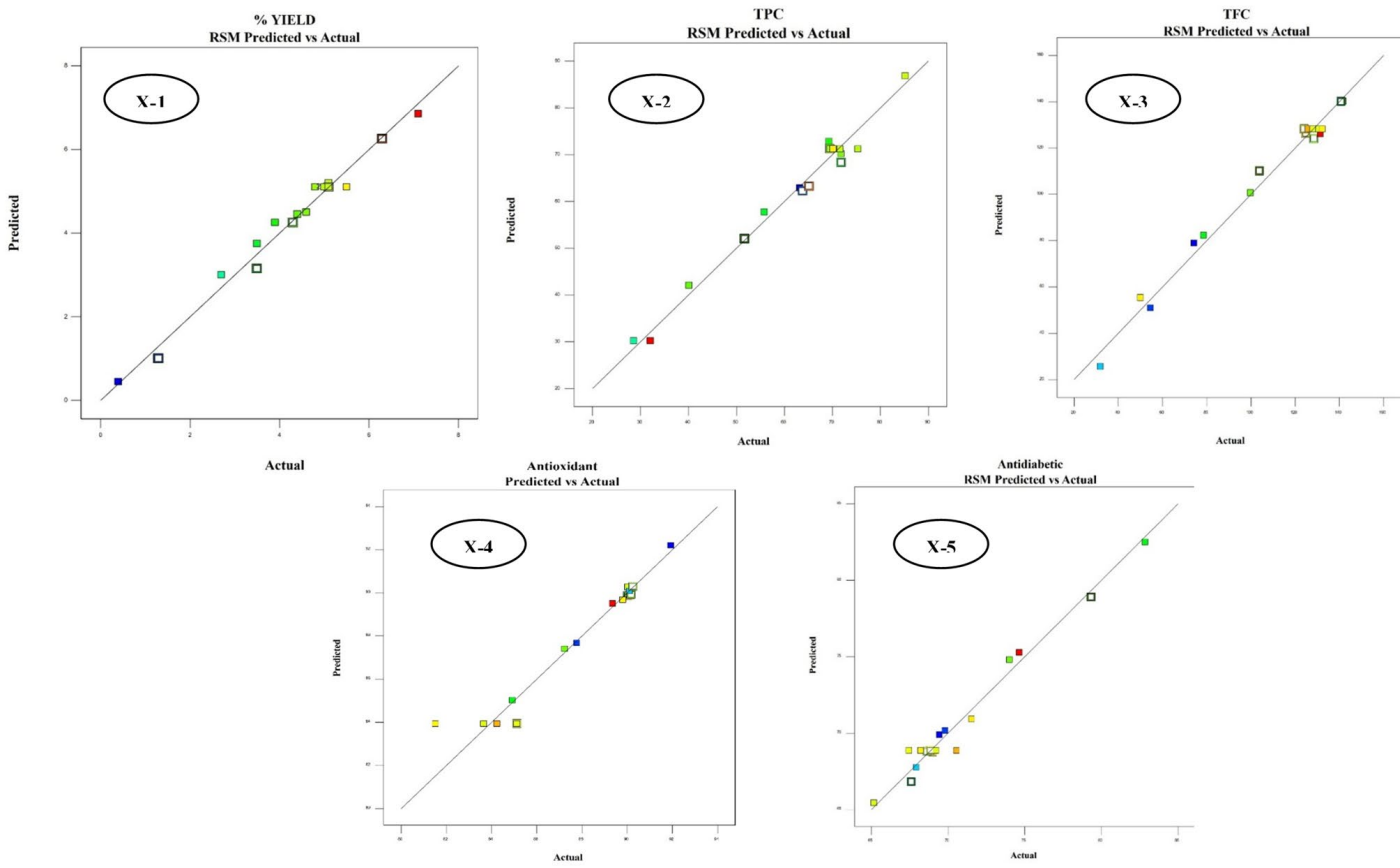
Graphs X-1 to X-5 are showing a correlations across all the predicted and actual values for %yield, TP and TF, antioxidant (AC) and antidiabetic (AD) activity of SNV seeds extract.

In Fig. 1, graphs from (a) to (c) show the percentage yield of SNV seeds in AA/PA DES. The DES/feed ratio (mL/g) (X_3_) has no significant effect on % yield, but a decrease in X_2_ (temperature) and an increase in X_1_ (ultrasound time) increases the extraction yield. This justify the increase in mass transfer and plant cell rupture during the ultrasound treatment. In a previous study, Naseem, et al. ^40^ have observed ultrasound treatment can improve the mass transfer during DES-UAE based extraction of phytochemical from *Mentha arvensis* leaves. An increase in temperature may also increase the mass transfer but it deteriorates the phytochemicals. The similar kind of temperature effect has been documented by Chen, et al. ^41^, who undertaken enzyme-ultrasound assisted of phenolics from *Epimedium* leaves. The graphs from (d) to (f) show that X_1_, X_2_, and X_3_ are affecting positively on total phenolic content. The maximum increase in TP value was observed for prolonged time of sonication. Wu, et al. ^42^ also noted an increase in TP of *Moringa oleifera* L extracts produced under prolonged extraction conditions of DES-UAE. The temperature increase increases the TP up to the middle as shown in graph (d), and then decreases with the increase in temperature because heating at high temperature deteriorates the phenolic content of the plant. Anila and Farid reported the same effect of temperature increase up to optimal value on total phenolic contents of red cabbage, canola seeds and black currants^43^. The graphs (g) to (i) show the effect of X_1_, X_2_, and X_3_ on total flavonoid content. Interestingly, time and temperature have no significant effect on the total flavonoid content, but the DES/feed ratio is affecting positively the TF values because the solvent increase facilitates the extraction of bioactives, resulting in enhanced overall value as previously reported by Rashid, et al. ^44^ on apple pomace ultrasound assisted extraction in which solvent increase impacted positively on the yield of total flavonoids. The effect of time, temperature, and DES/feed ratio on the antioxidant potential of SNV seed extract are shown in graphs (j) to (l). It can be observed that the temperature increase decreases the DPPH inhibitory activity because prolonged heating can deteriorate the phytochemicals. This effect is previously reported by Airouyuwa et al., (2023) on *Phoenix dactylifera L*. extraction in which high temperature degraded the phytochemicals^45^. Time and solvent ratio increase is overall increasing the antioxidant (AC) potential because optimal sonication time and solvent help is better washing of phytochemicals that are responsible for antioxidant activity as previously reported by Rashid et al., (2023)^44^. The antidiabetic potential of SNV seed and the effects of X_1_, X_2_, and X_3_ are shown in graphs (m) to (o). It can be seen that increase in extraction time decreases the antidiabetic activity because prolonged heating can denature the phytochemicals, while the increase in DES (solvent) and operating temperature increase the antidiabetic potential because both factors help in better draining of bioactives that are effecting the antidiabetic activity in less time as previously reported by Iftikhar et al. (2025) on the extraction of *Strychnos potatorum* L. seeds.

In Fig. 2, the graphs compare predicted and actual values of % yield, TP and TF, AC, and AD. A strong linear correlation is overall seen in all graphs from (X-1 to X-5). The model for %yield (Graph X-1) shows reasonable precision, although minor deviations occur at lower values. TP (Graph X-2) and TF (Graph X-3) exhibit robust predictive performance with most points tightly clustered, though slight variability is observed at the extremes. The AC model (Graph X-4) demonstrates excellent precision, with minimal deviations and strong predictive accuracy. Similarly, the AD model (Graph X-5) aligns well with actual values, although minor variability exists in the lower range. Overall, the RSM models are reliable, with high predictive accuracy for all responses, particularly antioxidant and TP. However, slight improvements could enhance precision at extreme response ranges, especially for %yield.

### Artificial neural networking (ANN)

RSM and ANN were used to develop predictive models based on the output data. The generated model equations were further refined and theoretically optimized, and their accuracy and suitability were examined using statistical tools of SPSS in validation and acceptance. In the case of the ANN approach, layer weights and biases were selected according to the value of the MSE of the LM algorithm^12^.

The dataset used in ANN was partitioned into three subsets: training (70%), testing (15%), and validation (15%). We prioritized training results based on MSE values and the correlation coefficients (R) between actual and predicted values. The methodology described here is implemented in MATLAB (R2019a) with the standard feature of ANN analysis. Furthermore, ANN models were implemented to analyze data from the ultrasound-assisted extraction (UAE) process, focusing on five output parameters: yield (%), TP and TF, AC, and AD potential.

The R-value obtained for % yield, TP and TF, AC, and AD activity is greater than 0.99 for testing training and validation in all dependent variables. The value of R for all datasets is > 0.99 except for antioxidant activity which is 0.96851. For % yield, the best validation performance (BVP) is 1.1116e-23 at epoch 0, for TP, BVP is 6.4951 at epoch 1, for TF, BVP is 9.5943 at epoch 2, for AC, BVP is 3.2777 at epoch 0, and for AD, BVP is 0.78956 at epoch 0. The results are shown in ***Figure S2F2*** and ***Figure S2F3***.

### Results based on ANN and RSM optimization

The experimental results obtained by using optimization conditions predicted by RSM give the maximum % yield (6.31%), TP (64.72 mg GAE/g), TF (113.19 mg QE/g), AC (89.87%), and AD (68.53%) activity at time of 25 min, 40 ℃ temperature, and DES/feed ratio of 30 mL/g. The coefficient of variation (%RSD) shows that ANN shows consistently lower %RSD, which shows its higher accuracy and closer alignment with the experimental data. Overall, both methodologies provide reliable predictions. It can be seen that DES-UAE extraction is showing better results than control (ethanol) in all responses except TF. Table 5 shows the experimental values along with RSM and ANN predicted values.

**Table 5:** Actual experimental values vs. RSM-predicted and ANN-predicted values using RSM suggested optimal conditions and their %RSD.

Dependent Variables	Experimental values	RSM-predicted results	% RSD-RSM	ANN-Predicted results	% RSD-ANN	Control (Ethanol)
% Yield	6.31 ± 0.04	6.25	0.68	6.30	0.11	4.98 ± 0.42
TP	64.72 ± 0.01	63.23	1.65	65.19	0.51	59.14 ± 0.81
TF	103.19 ± 0.08	110.06	4.56	104.57	0.94	122.14 ± 0.91
Antioxidant activity	89.87 ± 0.02	90.28	0.32	90.25	0.29	78.14 ± 0.15
Antidiabetic activity	68.53 ± 0.07	68.80	0.28	68.49	0.04	59.51 ± 0.45

Results of a comparative prediction study using RSM vs. ANN for predicting the extraction outcome show that ANN consistently outperforms RSM for all parameters. Results show that the ANN approach outperforms by achieving lower AAD, PPE, % RSD and RMSE and higher R^2^ values. ANN achieved an AAD of 1.70%, much improved than the RSM’s 4.72% and with an improved R^2^ value of 0.9885 for the % yield. ANN has also been shown to perform better with the less PPE and high R² values of TP and TF: 0.9890 and 0.9963, respectively. This trend holds for antioxidant and antidiabetic activities, where ANN results in the lowest RMSE and highest R² values, suggesting its robust prediction. The results presented here confirm that ANN is capable of relatively reliable capturing of the complex relationships contained in the data and hence, is a better predictive tool to RSM for the tasks of extracting process parameter optimums. Therefore, the combination of RSM with ANN analysis is preferred for precise modeling studies for the multifactorial optimization of bioactive compounds.

(Refer to supplementary data file **S1D11** for RSM predicted values, ***S1Y1 to S1Y6*** for % yield, ***S1P1 to S1P6*** for TP, ***S1F1 to S1F6*** for TF, ***S1D1 to S1D6*** for AC, and ***S1α1 to S1α6*** for AD. ***Figure S2F1*** showing the ANN architecture and hidden no of neurons) Table 6.

**Table 6:** %AAD, PPE, RMSE, and R² for % yield, TP and TF, DPPH-RSC, and AD activity as predicted by RSM and ANN models.

Parameters	% Yield		TP		TF		Antioxidant (AC)		Antidiabetic (AD)	
Technique used	RSM-values	ANN-values	RSM-values	ANN-values	RSM-values	ANN-values	RSM-values	ANN-values	RSM-values	ANN-values
AAD	4.72	1.70	2.52	1.26	3.60	1.15	0.40	0.37	0.63	0.32
PPE	9.02	7.57	2.29	0.69	3.12	2.08	0.97	0.94	0.78	0.56
RMSE	0.22	0.18	2.09	1.60	3.87	2.08	0.73	0.74	0.70	0.57
R^2^	0.9810	0.9885	0.9809	0.9890	0.9870	0.9963	0.9382	0.9378	0.9749	0.9832

### Pearson’s correlation study

Pearson’s correlation analysis calculates the linear relationship between two variables^46^. Table 7 shows the correlations between % yield, TP, TF, AC, and AD activities. TP and TF show positive correlations (*r* = 0.638, *p* = 0.006). It reveals that the increase in TP also increases the TFC content significantly. The % yield shows weak correlations between other variables, and yield is negatively correlated with antidiabetic activity (*r* = -0.452, *p* = 0.068). It shows that the conditions that are increasing the % yield are decreasing the antidiabetic activity. Only weak correlations are present between antioxidant activity and other variables. Overall, TP and TF are strongly correlated, and other factors are not influenced by these variables.

**Table 7:** Pearson’s correlations between experimental values of % yield, TP and TF, AC, and AD activity.

Correlations						
		Experimental yield	Experimental TP	Experimental TF	Experimental AC	Experimental AD
**Experimental yield**	Pearson Correlation	1	0.038	-0.069	-0.248	-0.452
	Sig. (2-tailed)		0.884	0.793	0.337	0.068
	N	17	17	17	17	17
**Experimental TP**	Pearson Correlation	0.038	1	0.638^**^	-0.353	0.058
	Sig. (2-tailed)	0.884		0.006	0.165	0.826
	N	17	17	17	17	17
**Experimental TF**	Pearson Correlation	-0.069	0.638^**^	1	-0.286	-0.141
	Sig. (2-tailed)	0.793	0.006		0.266	0.590
	N	17	17	17	17	17
**Experimental AC**	Pearson Correlation	-0.248	-0.353	-0.286	1	-0.056
	Sig. (2-tailed)	0.337	0.165	0.266		0.832
	N	17	17	17	17	17
**Experimental AD**	Pearson Correlation	-0.452	0.058	-0.141	-0.056	1
	Sig. (2-tailed)	0.068	0.826	0.590	0.832	
	N	17	17	17	17	17

** Positive correlation.

### SEM-EDX analysis

**Fig. 3:**
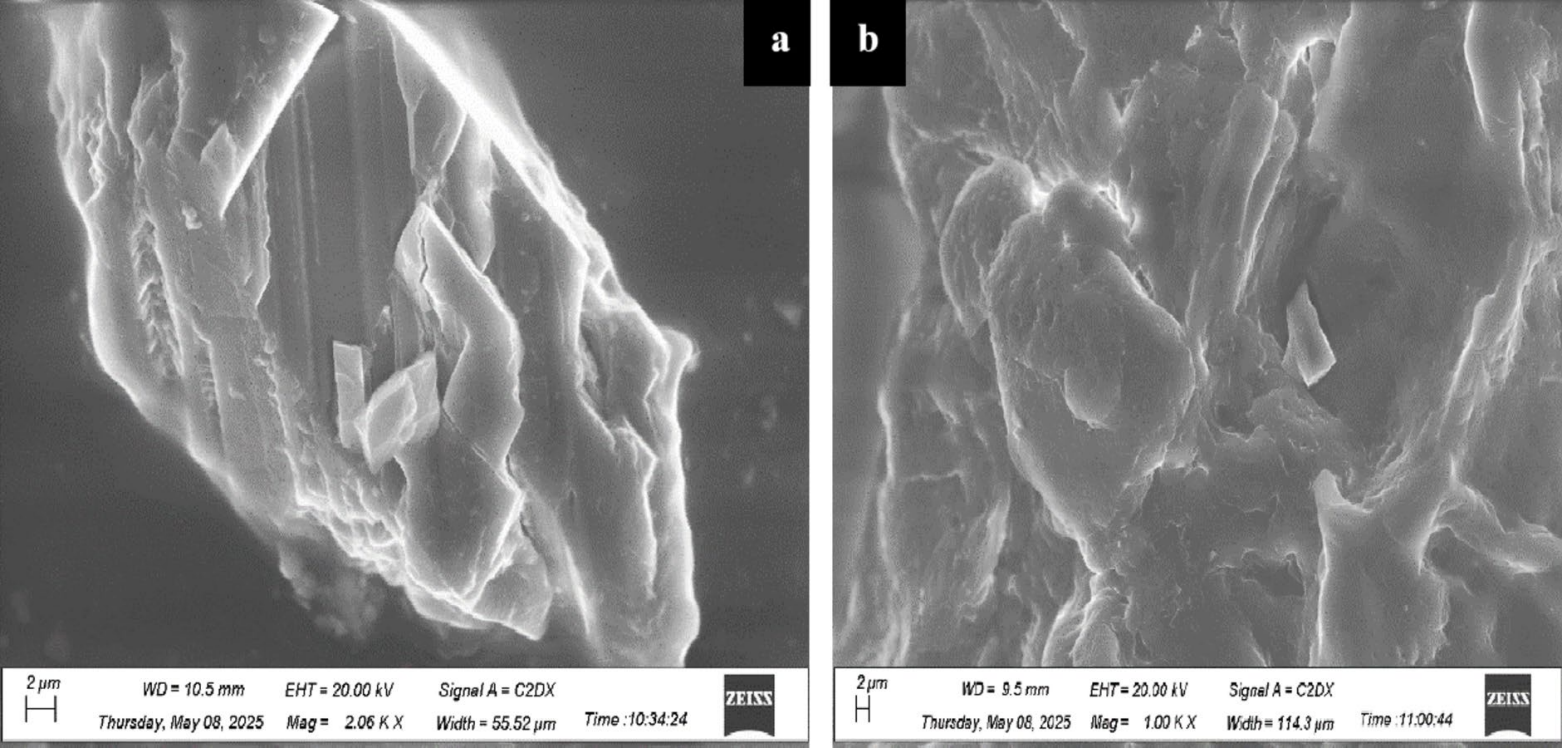
SEM (Scanning Electron Microscopy) generated images of *Strychnos nux-vomica* seed powder: (a) untreated control, showing a compact and crystalline surface morphology at 2.06 KX magnification with a field width of 55.52 μm; (b) sample after extraction using DES-assisted ultrasound extraction, displaying a disrupted and porous surface structure at 1.00 KX magnification with a field width of 114.3 μm. Scale bar of 2 μm is used in both images.

The comparative micrographs shown in Fig. 3 (a) and 3 (b) demonstrate the significant change in morphology between the untreated and treated SNV seeds, respectively. A significant structural alteration was induced by sonication-assisted extraction using a deep eutectic solvent. It can be identified in Fig. 3 (a) that the surface morphology is crystalline, dense and has a lamellar structure. The porosity is almost negligible, and a dense, rigid and intact matrix can be seen, indicating low porosity and less solvent penetration. These findings show that the untreated SNV seed powder indicates limited accessibility to bioactive compounds.

In Fig. 3 (b), obvious maceration, with the disappearance of angular crystals and a disordered, wrinkled, and eroded morphology, can be seen. This indicates a breakdown of the rigid cell wall, resulting in an amorphous sponge-like structure, which leads to better mass transfers when using the sonication technique coupled with DES^47,48^. This microstructural evolution clearly validates that sonication coupled with DES is a sustainable, viable, and green approach that enhances the phytochemical extraction from structurally rigid plant matrices.

The structural alteration was further investigated using energy-dispersive X-ray spectroscopy (EDX). Table 8 shows the change in carbon and oxygen percentages before and after extraction. Results indicate that oxygen and carbon are the major elements present in the seeds of SNV, as these elements are characteristic components of cellulose^49^. The untreated sample exhibits a higher carbon percentage (68.51% mass and 74.35% atoms), which indicates a dense lingocellulosic matrix (waxes, lipids and polysaccharides). The sample subjected to DES-UAE shows a decrease in carbon content (57.96% mass and 64.75% atoms), which indicates the recovery of plant bioactives or washing of carbon-rich hydrophobic layers. The increase in oxygen percentage, as shown in Table 8, from 31.49% (untreated) to 42.04% (treated), indicates the exposure of polar functional groups such as phenolics. The increase in surface oxygen indicates improved solubility and better diffusion of phytochemicals in DES.

The surface morphological and elemental percentage change confirms that sonication coupled with DES is a sustainable and green method for enhancing phytochemical extraction from complex plant matrices.

**Table 8:** Elemental composition of *Strychnos nux-vomica* L. seeds before (raw) and after extraction.

Element	Raw seed powder (Untreated)		Treated sample (Sonicated)	
	Mass %	Atom %	Mass %	Atom %
**Carbon**	68.51	74.35	57.96	64.75
**Oxygen**	31.49	25.65	42.04	35.25

## Conclusions

The results of the present research successfully highlighted that ammonium acetate and propanoic acid (AA/PA) DES coupled with ultrasound (DES-UAE) provides an efficient method for extracting phenolics from *Strychnos nux-vomica* L. (SNV) seeds. Albeit, AA/PA DES comes with high viscosity, careful selection of extraction conditions i.e. amounts of DES, ultrasound time, temperature, and solvent to feed ratio can help to get the recovery of bioactive compounds significantly (*p* ≤ 0.05) higher than conventional ethanol solvent based orbital shaking. It was further noticed that the bioactive extracts via optimized DES-UAE involving extraction time of 25 min at 40 °C and solvent to feed ratio of 30 mL/g offered better phenolics (TP) and flavonoids (TF) along with impressive antioxidant and antidiabetic activities. The application of mathematical and statistical tools like Response Surface Methodology (RSM) and Artificial Neural Networking (ANN) can facilitate the modulation of conditions with minimum number of experiments. It should be mentioned here that there was close agreement between the results observed in laboratory validation experiments and those predicted by the quadratic equations generated following RSM and ANN. Finally, the morphological and elemental change in SNV) seeds observed by SEM-EDX analysis indicates that AA/PA DES facilitated the maceration of seeds. These findings may provide a valuable DES framework to level up the extraction of secondary metabolites for nutraceutical and pharmaceutical applications.

### Scope and limitations of the study

This study suggests a sustainable and efficient extraction strategy using ammonium acetate–propanoic acid (AA/PA) DES coupled with ultrasound for recovering phenolics and flavonoids from *Strychnos nux-vomica* L. seeds. While the findings highlight the method’s efficiency, there are several limitations that should be acknowledged. First, the work focuses primarily on extraction optimization and in vitro antioxidant and antidiabetic assays; therefore, clinical relevance, bioavailability, and human safety aspects have not been evaluated and required future investigation. Although strychnine and brucine are known toxic alkaloids, this study did not quantify them in extracts produced by DES-UAE. The scalability of the DES-UAE method was not assessed beyond laboratory conditions, and factors such as solvent recovery, waste minimization, and process reproducibility under industrial settings need to be systematically studied. The toxicities of using ammonium acetate–propanoic acid (AA/PA) also could not be established. Ethical considerations and regulatory frameworks for handling toxic plant materials and translating these extracts into safe commercial products also need to be integrated into future work. Addressing these limitations in follow-up studies will help bridge the gap between laboratory-scale optimization and real-world clinical or industrial applications.

## Supplementary Information

Below is the link to the electronic supplementary material.

Supplementary Material 1

## Data Availability

The datasets generated and/or analyzed during the current study will be provided on request.
