# Supplementary material for: Optimization of phenolics recovery from strychnos nux-vomica L. seed applying deep eutectic solvent-ultrasound assisted extraction
Source: Sci Rep. 2026 Apr 17;16:17924. doi: 10.1038/s41598-026-47748-4 (PMC13249935; doi:10.1038/s41598-026-47748-4)
Supplement: Supplementary file 1 — Supplementary Material 1 [file 41598_2026_47748_MOESM1_ESM.docx]

**Supplementary data and supporting information**

The given data in this file shows the experimental design solution from RSM. The calculations for %PPE, %AAD, RMSE and R^2^ are given in this table for %yield, TPC, TFC, %DPPH and %antidiabetic activity of *Strychnos nux vomica* extract using the ultrasonication method.

1. **RSM-Design File Solutions**

***Table S1D11: Solutions from the design of expert predicted by RSM for %yield, TPC, TFC, % DPPH and % antidiabetic activity.***

| **Number** | **Time** | **Temperature** | **Solvent-Sample Ratio** | **% Yield UAE** | **TPC UAE** | **TFC UAE** | **Alpha-Amylase UAE** | **Anti-Oxidant UAE** | **Desirability** |
| --- | --- | --- | --- | --- | --- | --- | --- | --- | --- |
| 1 | *25* | 40 | 30 | 6.25 | 63.229 | 110.061 | 68.8 | 90.285 | 1 |
| 2 | *37.267* | 40 | 30 | 6.086 | 70.227 | 114.808 | 69.347 | 92.023 | 1 |
| 3 | *14.755* | 40 | 30 | 4.35 | 60.892 | 120.189 | 69.122 | 92.666 | 1 |
| 4 | *31.58* | 40 | 30 | 6.493 | 66.413 | 110.321 | 68.967 | 90.595 | 1 |
| 5 | *35.733* | 40 | 30 | 6.252 | 69.102 | 113.209 | 69.223 | 91.532 | 1 |
| 6 | *13.448* | 40 | 30 | 3.974 | 60.823 | 122.402 | 69.214 | 93.221 | 1 |
| 7 | *37.624* | 40 | 30 | 6.042 | 70.499 | 115.221 | 69.378 | 92.148 | 1 |
| 8 | *16.45* | 40 | 30 | 4.792 | 61.058 | 117.627 | 69.02 | 92.031 | 1 |
| 9 | *19.204* | 40 | 30 | 5.403 | 61.514 | 114.215 | 68.895 | 91.203 | 1 |
| 10 | *35.252* | 40 | 30 | 6.296 | 68.763 | 112.766 | 69.187 | 91.394 | 1 |
| 11 | *21.703* | 40 | 30 | 5.841 | 62.128 | 111.921 | 68.826 | 90.671 | 1 |
| 12 | *29.402* | 40 | 30 | 6.497 | 65.213 | 109.649 | 68.879 | 90.333 | 1 |
| 13 | *35.099* | 40 | 30 | 6.308 | 68.657 | 112.631 | 69.176 | 91.352 | 1 |
| 14 | *13.626* | 40 | 30 | 4.027 | 60.829 | 122.089 | 69.201 | 93.142 | 1 |
| 15 | *33.218* | 40 | 30 | 6.434 | 67.411 | 111.208 | 69.054 | 90.896 | 1 |
| 16 | *14.949* | 40 | 30 | 4.403 | 60.906 | 119.877 | 69.109 | 92.589 | 1 |
| 17 | *18.434* | 40 | 30 | 5.246 | 61.364 | 115.075 | 68.925 | 91.409 | 1 |
| 18 | *38.808* | 40 | 30 | 5.878 | 71.43 | 116.705 | 69.487 | 92.595 | 1 |
| 19 | *14.526* | 40 | 30 | 4.286 | 60.876 | 120.56 | 69.137 | 92.759 | 1 |
| 20 | *25.159* | 40 | 30 | 6.265 | 63.29 | 110.005 | 68.801 | 90.276 | 1 |
| 21 | *21.295* | 40 | 30 | 5.777 | 62.015 | 112.243 | 68.835 | 90.743 | 1 |
| 22 | *11.286* | 40 | 30 | 3.286 | 60.824 | 126.525 | 69.391 | 94.263 | 1 |
| 23 | *32.406* | 40 | 30 | 6.469 | 66.906 | 110.728 | 69.009 | 90.736 | 1 |
| 24 | *22.633* | 40 | 30 | 5.976 | 62.405 | 111.262 | 68.811 | 90.525 | 1 |
| 25 | *29.26* | 40 | 30 | 6.494 | 65.14 | 109.626 | 68.875 | 90.322 | 1 |
| 26 | *10.712* | 40 | 30 | 3.089 | 60.848 | 127.715 | 69.444 | 94.566 | 1 |
| 27 | *16.008* | 40 | 30 | 4.682 | 61.006 | 118.261 | 69.044 | 92.188 | 1 |
| 28 | *37.505* | 40 | 30 | 6.057 | 70.408 | 115.082 | 69.367 | 92.106 | 1 |
| 29 | *20.192* | 40 | 30 | 5.589 | 61.734 | 113.217 | 68.863 | 90.968 | 1 |
| 30 | *12.409* | 40 | 30 | 3.653 | 60.806 | 124.313 | 69.295 | 93.702 | 1 |
| 31 | *11.52* | 40 | 30 | 3.364 | 60.817 | 126.049 | 69.37 | 94.142 | 1 |
| 32 | *11.116* | 40 | 30 | 3.228 | 60.83 | 126.873 | 69.407 | 94.352 | 1 |
| 33 | *28.484* | 40 | 30 | 6.474 | 64.751 | 109.54 | 68.852 | 90.27 | 1 |
| 34 | *24.023* | 40 | 30 | 6.149 | 62.868 | 110.474 | 68.8 | 90.362 | 1 |
| 35 | *19.407* | 40 | 30 | 5.442 | 61.557 | 114.001 | 68.888 | 91.153 | 1 |
| 36 | *36.912* | 40 | 30 | 6.128 | 69.96 | 114.412 | 69.317 | 91.902 | 1 |
| 37 | *15.877* | 40 | 30 | 4.648 | 60.992 | 118.454 | 69.052 | 92.235 | 1 |
| 38 | *34.989* | 40 | 30 | 6.318 | 68.582 | 112.537 | 69.168 | 91.322 | 1 |
| 39 | *34.463* | 40 | 30 | 6.358 | 68.224 | 112.102 | 69.132 | 91.185 | 1 |
| 40 | *38.294* | 40 | 30 | 5.952 | 71.021 | 116.04 | 69.438 | 92.395 | 1 |
| 41 | *36.301* | 40 | 30 | 6.195 | 69.51 | 113.768 | 69.267 | 91.705 | 1 |
| 42 | *16.659* | 40 | 30 | 4.843 | 61.084 | 117.337 | 69.008 | 91.96 | 1 |
| 43 | *22.463* | 40 | 30 | 5.952 | 62.353 | 111.374 | 68.814 | 90.55 | 1 |
| 44 | *20.377* | 40 | 30 | 5.622 | 61.779 | 113.043 | 68.857 | 90.927 | 1 |
| 45 | *12.231* | 40 | 30 | 3.597 | 60.806 | 124.652 | 69.31 | 93.788 | 1 |
| 46 | *38.476* | 40 | 30 | 5.926 | 71.164 | 116.271 | 69.455 | 92.465 | 1 |
| 47 | *37.057* | 40 | 30 | 6.111 | 70.069 | 114.573 | 69.329 | 91.951 | 1 |
| 48 | *12.943* | 40 | 30 | 3.82 | 60.811 | 123.315 | 69.252 | 93.45 | 1 |
| 49 | *39.657* | 40 | 30 | 5.745 | 72.123 | 117.873 | 69.571 | 92.944 | 1 |
| 50 | *17.933* | 40 | 30 | 5.137 | 61.275 | 115.675 | 68.946 | 91.554 | 1 |
| 51 | *21.885* | 40 | 30 | 5.868 | 62.18 | 111.783 | 68.823 | 90.64 | 1 |
| 52 | *16.779* | 40 | 30 | 4.872 | 61.1 | 117.171 | 69.002 | 91.919 | 1 |
| 53 | *13.122* | 40 | 30 | 3.875 | 60.814 | 122.989 | 69.238 | 93.368 | 1 |
| 54 | *32.262* | 40 | 30 | 6.474 | 66.819 | 110.651 | 69.001 | 90.71 | 1 |
| 55 | *25.899* | 40 | 30 | 6.328 | 63.586 | 109.785 | 68.806 | 90.243 | 1 |
| 56 | *20.916* | 40 | 30 | 5.715 | 61.914 | 112.561 | 68.843 | 90.816 | 1 |
| 57 | *10.823* | 40 | 30 | 3.128 | 60.842 | 127.481 | 69.433 | 94.506 | 1 |
| 58 | *39.177* | 40 | 30 | 5.821 | 71.729 | 117.202 | 69.523 | 92.744 | 1 |
| 59 | *38.656* | 40 | 30 | 5.9 | 71.308 | 116.505 | 69.473 | 92.535 | 1 |
| 60 | *30.547* | 40 | 30 | 6.505 | 65.826 | 109.93 | 68.921 | 90.451 | 1 |
| 61 | *12.725* | 40 | 30 | 3.753 | 60.807 | 123.718 | 69.27 | 93.552 | 1 |
| 62 | *28.28* | 40 | 30 | 6.467 | 64.652 | 109.53 | 68.847 | 90.26 | 1 |
| 63 | *10.348* | 40 | 30 | 2.962 | 60.868 | 128.489 | 69.478 | 94.763 | 1 |
| 64 | *23.731* | 40 | 30 | 6.115 | 62.766 | 110.62 | 68.801 | 90.391 | 1 |
| 65 | *31.41* | 40 | 30 | 6.496 | 66.315 | 110.248 | 68.959 | 90.569 | 1 |
| 66 | *27.107* | 40 | 30 | 6.411 | 64.105 | 109.569 | 68.822 | 90.228 | 1 |
| 67 | *17.633* | 40 | 30 | 5.071 | 61.226 | 116.048 | 68.96 | 91.645 | 1 |
| 68 | *34.823* | 40 | 30 | 6.331 | 68.468 | 112.396 | 69.157 | 91.278 | 1 |
| 69 | *19.855* | 40 | 30 | 5.527 | 61.656 | 113.544 | 68.873 | 91.045 | 1 |
| 70 | *17.76* | 40 | 30 | 5.099 | 61.246 | 115.889 | 68.954 | 91.606 | 1 |
| 71 | *19.006* | 40 | 30 | 5.363 | 61.474 | 114.43 | 68.902 | 91.255 | 1 |
| 72 | *38.1* | 40 | 30 | 5.979 | 70.869 | 115.797 | 69.421 | 92.322 | 1 |
| 73 | *35.882* | 40 | 30 | 6.238 | 69.208 | 113.352 | 69.234 | 91.576 | 1 |
| 74 | *18.755* | 40 | 30 | 5.312 | 61.425 | 114.71 | 68.912 | 91.321 | 1 |
| 75 | *39.987* | 40 | 29.989 | 5.688 | 72.39 | 118.422 | 69.598 | 93.08 | 1 |
| 76 | *10* | 40 | 29.865 | 2.852 | 61.216 | 130.003 | 69.402 | 94.858 | 0.997 |
| 77 | *40* | 40 | 29.671 | 5.63 | 72.13 | 120.54 | 69.384 | 92.901 | 0.992 |
| 78 | *10* | 40 | 29.671 | 2.874 | 61.675 | 131.063 | 69.246 | 94.718 | 0.992 |
| 79 | *40* | 40 | 29.445 | 5.591 | 71.923 | 121.974 | 69.236 | 92.774 | 0.986 |
| 80 | *10* | 40 | 29.114 | 2.937 | 62.94 | 133.914 | 68.817 | 94.326 | 0.978 |
| 81 | *39.998* | 40.512 | 30 | 5.646 | 74.865 | 120.546 | 70.249 | 92.568 | 0.974 |
| 82 | *40* | 40 | 28.29 | 5.397 | 70.667 | 128.564 | 68.546 | 92.165 | 0.956 |
|  |  |  |  |  |  |  |  |  |  |

1. **Yield_AAD_UAE**

***Table: S1Y1: Table shows the percentage absolute average deviation for (%AAD) for %yield.***

| **Yield_AAD_UAE** |  |  |  |  |
| --- | --- | --- | --- | --- |
| Experimental | RSM_PREDICTED | RSM_AAD | ANN_PREDICTED | ANN_AAD |
| 3.5 | 3.15 | 0.1000000 | 3.5000000 | 0.0000000 |
| 5.1 | 5.2 | 0.0196078 | 5.1000000 | 0.0000000 |
| 4.6 | 4.5 | 0.0217391 | 4.6000000 | 0.0000000 |
| 4.3 | 4.25 | 0.0116279 | 4.3000000 | 0.0000000 |
| 4.8 | 5.1 | 0.0625000 | 5.0500000 | 0.0520833 |
| 5.1 | 5.1 | 0.0000000 | 5.0500000 | 0.0098039 |
| 3.9 | 4.25 | 0.0897436 | 3.9000000 | 0.0000000 |
| 1.3 | 1 | 0.2307692 | 1.1837703 | 0.0894075 |
| 5.5 | 5.1 | 0.0727273 | 5.0500000 | 0.0818182 |
| 0.4 | 0.45 | 0.1250000 | 0.4000000 | 0.0000000 |
| 2.7 | 3 | 0.1111111 | 2.6473772 | 0.0194899 |
| 7.1 | 6.85 | 0.0352113 | 7.1000000 | 0.0000000 |
| 4.4 | 4.45 | 0.0113636 | 4.4000000 | 0.0000000 |
| 5.1 | 5.1 | 0.0000000 | 5.0500000 | 0.0098039 |
| 6.3 | 6.25 | 0.0079365 | 5.7686646 | 0.0843390 |
| 5 | 5.1 | 0.0200000 | 5.0500000 | 0.0100000 |
| 3.5 | 3.75 | 0.0714286 | 3.5000000 | 0.0000000 |
| **%AAD** |  | 4.72 |  | 1.70 |

***Table S1Y2: Calculated values for the percentage prediction error (%PPE) for %yield.***

| **PPE_YIELD_UAE** |  |  |  |  |
| --- | --- | --- | --- | --- |
| EXPERIMENTAL | RSM_OPTIMIZED | PPE_RSM | ANN_OPTIMIZED | PPE_ANN |
| 6.87 | 6.25 | 9.02 | 6.35 | 7.57 |

***Table S1Y3: Calculations of the Root mean square velocity (RMSE) for RSM %yield.***

| **YIELD_RMSE_RSM_ UAE** |  |  |  |
| --- | --- | --- | --- |
| EXPERIMENTAL | RSM_PREDICTED | Errors_RSM | Square |
| 3.5 | 3.15 | 0.350 | 0.123 |
| 5.1 | 5.2 | -0.100 | 0.010 |
| 4.6 | 4.5 | 0.100 | 0.010 |
| 4.3 | 4.25 | 0.050 | 0.002 |
| 4.8 | 5.1 | -0.300 | 0.090 |
| 5.1 | 5.1 | 0.000 | 0.000 |
| 3.9 | 4.25 | -0.350 | 0.123 |
| 1.3 | 1 | 0.300 | 0.090 |
| 5.5 | 5.1 | 0.400 | 0.160 |
| 0.4 | 0.45 | -0.050 | 0.003 |
| 2.7 | 3 | -0.300 | 0.090 |
| 7.1 | 6.85 | 0.250 | 0.063 |
| 4.4 | 4.45 | -0.050 | 0.002 |
| 5.1 | 5.1 | 0.000 | 0.000 |
| 6.3 | 6.25 | 0.050 | 0.002 |
| 5 | 5.1 | -0.100 | 0.010 |
| 3.5 | 3.75 | -0.250 | 0.063 |
|  |  | MEAN/Average | 0.049 |
|  |  | RMSE | 0.222 |

***Table S1Y4: Calculations of the Root mean square velocity (RMSE) for ANN %yield.***

| **YIELD_RMSE_ANN_UAE** |  |  |  |
| --- | --- | --- | --- |
| EXPERIMENTAL | ANN_PREDICTED | Errors_RSM | Square |
| 3.5 | 3.5000000 | 0.000 | 0.000 |
| 5.1 | 5.1000000 | 0.000 | 0.000 |
| 4.6 | 4.6000000 | 0.000 | 0.000 |
| 4.3 | 4.3000000 | 0.000 | 0.000 |
| 4.8 | 5.0500000 | -0.250 | 0.062 |
| 5.1 | 5.0500000 | 0.050 | 0.003 |
| 3.9 | 3.9000000 | 0.000 | 0.000 |
| 1.3 | 1.1837703 | 0.116 | 0.014 |
| 5.5 | 5.0500000 | 0.450 | 0.203 |
| 0.4 | 0.4000000 | 0.000 | 0.000 |
| 2.7 | 2.6473772 | 0.053 | 0.003 |
| 7.1 | 7.1000000 | 0.000 | 0.000 |
| 4.4 | 4.4000000 | 0.000 | 0.000 |
| 5.1 | 5.0500000 | 0.050 | 0.003 |
| 6.3 | 5.7686646 | 0.531 | 0.282 |
| 5 | 5.0500000 | -0.050 | 0.002 |
| 3.5 | 3.5000000 | 0.000 | 0.000 |
|  |  | MEAN/Average | 0.034 |
|  |  | RMSE | 0.183 |

***Graph S1Y5: R^2^ values for RSM-predicted % yield.***

***Graph S1Y6: R^2^ values for ANN-predicted % yield.***

1. **Total Phenolic Content**

***Table: S1P1: Table shows the percentage absolute average deviation for (%AAD) for TPC.***

| **TPC_AAD_UAE** |  |  |  |  |
| --- | --- | --- | --- | --- |
| Experimental | RSM_PREDICTED | RSM_AAD | ANN_PREDICTED | ANN_AAD |
| 71.83 | 68.32 | 0.0488654 | 71.8350000 | 0.0000696 |
| 85.23 | 86.85 | 0.0190074 | 85.2260000 | 0.0000469 |
| 71.79 | 70.16 | 0.0227051 | 68.9490916 | 0.0395725 |
| 51.72 | 52.03 | 0.0059938 | 51.7211000 | 0.0000213 |
| 69.47 | 71.22 | 0.0251907 | 71.4452333 | 0.0284329 |
| 69.52 | 71.22 | 0.0244534 | 71.4452333 | 0.0276932 |
| 69.29 | 72.81 | 0.0508010 | 69.2903000 | 0.0000043 |
| 63.82 | 62.23 | 0.0249138 | 66.0496113 | 0.0349359 |
| 70.16 | 71.22 | 0.0151083 | 71.4452333 | 0.0183186 |
| 63.22 | 62.91 | 0.0049035 | 65.5302098 | 0.0365424 |
| 28.6 | 30.18 | 0.0552448 | 29.3216806 | 0.0252336 |
| 32.09 | 30.19 | 0.0592085 | 32.0866000 | 0.0001060 |
| 40.13 | 42.06 | 0.0480937 | 40.1269000 | 0.0000772 |
| 71.62 | 71.22 | 0.0055850 | 71.4452333 | 0.0024402 |
| 65.16 | 63.23 | 0.0296194 | 65.1581000 | 0.0000292 |
| 75.35 | 71.22 | 0.0548109 | 71.4452333 | 0.0518217 |
| 55.85 | 57.75 | 0.0340197 | 55.8537000 | 0.0000662 |
| **%AAD** |  | 2.52 |  | 1.26 |

***Table S1P2: Calculated values for the percentage prediction error (%PPE) for TPC.***

| **PPE_TPC** |  |  |  |  |
| --- | --- | --- | --- | --- |
| EXPERIMENTAL | RSM_OPTIMIZED | PPE_RSM | ANN_OPTIMIZED | PPE_ANN |
| 64.71 | 63.23 | 2.29 | 65.16 | 0.69 |

***Table S1P3: Calculations of the Root mean square velocity (RMSE) for RSM TPC.***

| **TPC_RMSE_RSM_UAE** |  |  |  |
| --- | --- | --- | --- |
| EXPERIMENTAL | RSM_PREDICTED | Errors_RSM | Square |
| 71.83 | 68.32 | 3.510 | 12.320 |
| 85.23 | 86.85 | -1.620 | 2.624 |
| 71.79 | 70.16 | 1.630 | 2.657 |
| 51.72 | 52.03 | -0.310 | 0.096 |
| 69.47 | 71.22 | -1.750 | 3.063 |
| 69.52 | 71.22 | -1.700 | 2.890 |
| 69.29 | 72.81 | -3.520 | 12.390 |
| 63.82 | 62.23 | 1.590 | 2.528 |
| 70.16 | 71.22 | -1.060 | 1.124 |
| 63.22 | 62.91 | 0.310 | 0.096 |
| 28.6 | 30.18 | -1.580 | 2.496 |
| 32.09 | 30.19 | 1.900 | 3.610 |
| 40.13 | 42.06 | -1.930 | 3.725 |
| 71.62 | 71.22 | 0.400 | 0.160 |
| 65.16 | 63.23 | 1.930 | 3.725 |
| 75.35 | 71.22 | 4.130 | 17.057 |
| 55.85 | 57.75 | -1.900 | 3.610 |
|  |  | MEAN/Average | 4.363 |
|  |  | RMSE | 2.09 |

***Table S1P4: Calculations of the Root mean square velocity (RMSE) for ANN TPC.***

| **TPC_RMSE_ANN_UAE** |  |  |  |
| --- | --- | --- | --- |
| EXPERIMENTAL | ANN_PREDICTED | Errors_RSM | Square |
| 71.83 | 71.8350000 | -0.005 | 0.000 |
| 85.23 | 85.2260000 | 0.004 | 0.000 |
| 71.79 | 68.9490916 | 2.841 | 8.071 |
| 51.72 | 51.7211000 | -0.001 | 0.000 |
| 69.47 | 71.4452333 | -1.975 | 3.902 |
| 69.52 | 71.4452333 | -1.925 | 3.707 |
| 69.29 | 69.2903000 | 0.000 | 0.000 |
| 63.82 | 66.0496113 | -2.230 | 4.971 |
| 70.16 | 71.4452333 | -1.285 | 1.652 |
| 63.22 | 65.5302098 | -2.310 | 5.337 |
| 28.6 | 29.3216806 | -0.722 | 0.521 |
| 32.09 | 32.0866000 | 0.003 | 0.000 |
| 40.13 | 40.1269000 | 0.003 | 0.000 |
| 71.62 | 71.4452333 | 0.175 | 0.031 |
| 65.16 | 65.1581000 | 0.002 | 0.000 |
| 75.35 | 71.4452333 | 3.905 | 15.247 |
| 55.85 | 55.8537000 | -0.004 | 0.000 |
|  |  | MEAN/Average | 2.555 |
|  |  | RMSE | 1.60 |

***Graph S1P5: R^2^ values for RSM-predicted TPC.***

***Graph S1P6: R^2^ values for ANN-predicted TPC.***

1. **Total Flavonoid Content**

***Table: S1F1: Table shows the percentage absolute average deviation for (%AAD) for TFC.***

| **TFC_AAD_SHA** |  |  |  |  |
| --- | --- | --- | --- | --- |
| Experimental | RSM_PREDICTED | RSM_AAD | ANN_PREDICTED | ANN_AAD |
| 124.77 | 126.4 | 0.0130640 | 124.7329523 | 0.0002969 |
| 131.43 | 126.06 | 0.0408583 | 131.5927746 | 0.0012385 |
| 49.99 | 55.35 | 0.1072214 | 54.4202016 | 0.0886218 |
| 140.82 | 139.98 | 0.0059651 | 140.8217081 | 0.0000121 |
| 130.5 | 128.16 | 0.0179310 | 128.6720755 | 0.0140071 |
| 124.2 | 128.16 | 0.0318841 | 128.6720755 | 0.0360070 |
| 141.78 | 140.14 | 0.0115672 | 141.7609845 | 0.0001341 |
| 128.54 | 123.97 | 0.0355531 | 128.5222003 | 0.0001385 |
| 128.01 | 128.16 | 0.0011718 | 128.6720755 | 0.0051721 |
| 99.78 | 100.62 | 0.0084185 | 99.7521306 | 0.0002793 |
| 74.31 | 78.88 | 0.0614991 | 71.4171157 | 0.0389299 |
| 54.61 | 50.89 | 0.0681194 | 54.6054872 | 0.0000826 |
| 31.82 | 25.61 | 0.1951603 | 31.8162465 | 0.0001180 |
| 132.29 | 128.16 | 0.0312193 | 128.6720755 | 0.0273484 |
| 103.86 | 110.06 | 0.0596957 | 104.5067213 | 0.0062269 |
| 125.8 | 128.16 | 0.0187599 | 128.6720755 | 0.0228305 |
| 78.51 | 82.24 | 0.0475099 | 78.4960854 | 0.0001772 |
| **%AAD** |  | 3.60 |  | 1.15 |

***Table S1F2: Calculated values for the percentage prediction error (%PPE) for TFC.***

| **PPE_TFC** |  |  |  |  |
| --- | --- | --- | --- | --- |
| **EXPERIMENTAL** | **RSM_OPTIMIZED** | **PPE_RSM** | **ANN_OPTIMIZED** | **PPE_ANN** |
| 106.73 | 110.06 | 3.12 | 104.51 | 2.08 |

***Table S1F3: Calculations of the Root mean square velocity (RMSE) for RSM TFC.***

| **TFC_RMSE_RSM_UAE** |  |  |  |
| --- | --- | --- | --- |
| **EXPERIMENTAL** | **RSM_PREDICTED** | **Errors_RSM** | **Square** |
| 124.77 | 126.4 | -1.630 | 2.657 |
| 131.43 | 126.06 | 5.370 | 28.837 |
| 49.99 | 55.35 | -5.360 | 28.730 |
| 140.82 | 139.98 | 0.840 | 0.706 |
| 130.5 | 128.16 | 2.340 | 5.476 |
| 124.2 | 128.16 | -3.960 | 15.682 |
| 141.78 | 140.14 | 1.640 | 2.690 |
| 128.54 | 123.97 | 4.570 | 20.885 |
| 128.01 | 128.16 | -0.150 | 0.023 |
| 99.78 | 100.62 | -0.840 | 0.706 |
| 74.31 | 78.88 | -4.570 | 20.885 |
| 54.61 | 50.89 | 3.720 | 13.838 |
| 31.82 | 25.61 | 6.210 | 38.564 |
| 132.29 | 128.16 | 4.130 | 17.057 |
| 103.86 | 110.06 | -6.200 | 38.440 |
| 125.8 | 128.16 | -2.360 | 5.570 |
| 78.51 | 82.24 | -3.730 | 13.913 |
|  |  | MEAN/Average | 14.980 |
|  |  | RMSE | 3.87 |

***Table S1F4: Calculations of the Root mean square velocity (RMSE) for ANN TFC.***

| **TFC_RMSE_ANN_UAE** |  |  |  |
| --- | --- | --- | --- |
| **EXPERIMENTAL** | **ANN_PREDICTED** | **Errors_RSM** | **Square** |
| 124.77 | 124.7329523 | 0.037 | 0.001 |
| 131.43 | 131.5927746 | -0.163 | 0.026 |
| 49.99 | 54.4202016 | -4.430 | 19.627 |
| 140.82 | 140.8217081 | -0.002 | 0.000 |
| 130.5 | 128.6720755 | 1.828 | 3.341 |
| 124.2 | 128.6720755 | -4.472 | 19.999 |
| 141.78 | 141.7609845 | 0.019 | 0.000 |
| 128.54 | 128.5222003 | 0.018 | 0.000 |
| 128.01 | 128.6720755 | -0.662 | 0.438 |
| 99.78 | 99.7521306 | 0.028 | 0.001 |
| 74.31 | 71.4171157 | 2.893 | 8.369 |
| 54.61 | 54.6054872 | 0.005 | 0.000 |
| 31.82 | 31.8162465 | 0.004 | 0.000 |
| 132.29 | 128.6720755 | 3.618 | 13.089 |
| 103.86 | 104.5067213 | -0.647 | 0.418 |
| 125.8 | 128.6720755 | -2.872 | 8.249 |
| 78.51 | 78.4960854 | 0.014 | 0.000 |
|  |  | MEAN/Average | 4.327 |
|  |  | RMSE | 2.08 |

***Graph S1F5: R^2^ values for RSM-predicted TFC.***

***Graph S1F6: R^2^ values for ANN-predicted TFC.***

1. **Antioxidant Activity (DPPH-RSC)**

***Table: S1D1: Table shows the percentage absolute average deviation for (%AAD) for % DPPH.***

| **DPPH_AAD_UAE** |  |  |  |  |
| --- | --- | --- | --- | --- |
| **Experimental** | **RSM_PREDICTED** | **RSM_AAD** | **ANN_PREDICTED** | **ANN_AAD** |
| 90.11 | 89.88 | 0.0025524 | 89.8173902 | 0.0032473 |
| 89.36 | 89.49 | 0.0014548 | 89.3600000 | 0.0000000 |
| 89.81 | 89.68 | 0.0014475 | 89.6890552 | 0.0013467 |
| 90.03 | 89.87 | 0.0017772 | 90.0300000 | 0.0000000 |
| 83.66 | 83.93 | 0.0032273 | 83.8550000 | 0.0023309 |
| 85.13 | 83.93 | 0.0140961 | 83.8550000 | 0.0149771 |
| 90.03 | 90.26 | 0.0025547 | 89.8472773 | 0.0020296 |
| 90.18 | 89.92 | 0.0028831 | 90.1800000 | 0.0000000 |
| 85.11 | 83.93 | 0.0138644 | 83.8550000 | 0.0147456 |
| 87.24 | 87.4 | 0.0018340 | 87.2400000 | 0.0000000 |
| 91.94 | 92.2 | 0.0028279 | 91.8402334 | 0.0010851 |
| 87.77 | 87.67 | 0.0011393 | 88.2651405 | 0.0056413 |
| 90.11 | 90.09 | 0.0002220 | 90.1100000 | 0.0000000 |
| 81.52 | 83.93 | 0.0295633 | 83.8550000 | 0.0286433 |
| 90.26 | 90.29 | 0.0003324 | 90.2600000 | 0.0000000 |
| 84.24 | 83.93 | 0.0036800 | 83.8550000 | 0.0045703 |
| 84.92 | 85.02 | 0.0011776 | 84.9200000 | 0.0000000 |
| **%AAD** |  | 0.40 |  | 0.37 |

***Table S1D2: Calculated values for the percentage prediction error (%PPE) for % DPPH.***

| **PPE_DPPH** |  |  |  |  |
| --- | --- | --- | --- | --- |
| **EXPERIMENTAL** | **RSM_OPTIMIZED** | **PPE_RSM** | **ANN_OPTIMIZED** | **PPE_ANN** |
| 89.42 | 90.29 | 0.97 | 90.26 | 0.94 |

***Table S1D3: Calculations of the Root mean square velocity (RMSE) for RSM % DPPH.***

| **DPPH_RMSE_RSM_UAE** |  |  |  |
| --- | --- | --- | --- |
| **EXPERIMENTAL** | **RSM_PREDICTED** | **Errors_RSM** | **Square** |
| 90.11 | 89.88 | 0.230 | 0.053 |
| 89.36 | 89.49 | -0.130 | 0.017 |
| 89.81 | 89.68 | 0.130 | 0.017 |
| 90.03 | 89.87 | 0.160 | 0.026 |
| 83.66 | 83.93 | -0.270 | 0.073 |
| 85.13 | 83.93 | 1.200 | 1.440 |
| 90.03 | 90.26 | -0.230 | 0.053 |
| 90.18 | 89.92 | 0.260 | 0.068 |
| 85.11 | 83.93 | 1.180 | 1.392 |
| 87.24 | 87.4 | -0.160 | 0.026 |
| 91.94 | 92.2 | -0.260 | 0.068 |
| 87.77 | 87.67 | 0.100 | 0.010 |
| 90.11 | 90.09 | 0.020 | 0.000 |
| 81.52 | 83.93 | -2.410 | 5.808 |
| 90.26 | 90.29 | -0.030 | 0.001 |
| 84.24 | 83.93 | 0.310 | 0.096 |
| 84.92 | 85.02 | -0.100 | 0.010 |
|  |  | MEAN/Average | 0.539 |
|  |  | RMSE | 0.73 |

***Table S1D4: Calculations of the Root mean square velocity (RMSE) for ANN % DPPH.***

| **DPPH_RMSE_ANN_UAE** |  |  |  |
| --- | --- | --- | --- |
| **EXPERIMENTAL** | **ANN_PREDICTED** | **Errors_RSM** | **Square** |
| *90.11* | 89.8173902 | 0.293 | 0.086 |
| 89.36 | 89.3600000 | 0.000 | 0.000 |
| 89.81 | 89.6890552 | 0.121 | 0.015 |
| *90.03* | 90.0300000 | 0.000 | 0.000 |
| 83.66 | 83.8550000 | -0.195 | 0.038 |
| *85.13* | 83.8550000 | 1.275 | 1.626 |
| 90.03 | 89.8472773 | 0.183 | 0.033 |
| *90.18* | 90.1800000 | 0.000 | 0.000 |
| 85.11 | 83.8550000 | 1.255 | 1.575 |
| 87.24 | 87.2400000 | 0.000 | 0.000 |
| 91.94 | 91.8402334 | 0.100 | 0.010 |
| 87.77 | 88.2651405 | -0.495 | 0.245 |
| 90.11 | 90.1100000 | 0.000 | 0.000 |
| 81.52 | 83.8550000 | -2.335 | 5.452 |
| *90.26* | 90.2600000 | 0.000 | 0.000 |
| 84.24 | 83.8550000 | 0.385 | 0.148 |
| 84.92 | 84.9200000 | 0.000 | 0.000 |
|  |  | MEAN/Average | 0.543 |
|  |  | RMSE | 0.74 |

***Graph S1D5: R^2^ values for RSM-predicted % DPPH.***

***Graph S1D6: R^2^ values for ANN-predicted % DPPH.***

1. **Alpha-Amylase Inhibitory Activity**

***Table: S1α1: Table shows the percentage absolute average deviation for (%AAD) for % antidiabetic activity.***

| **Alpha_AAD_UAE** |  |  |  |  |
| --- | --- | --- | --- | --- |
| **Experimental** | **RSM_PREDICTED** | **RSM_AAD** | **ANN_PREDICTED** | **ANN_AAD** |
| 69.01 | 68.74 | 0.0039125 | 69.0825234 | 0.0010509 |
| 74.65 | 75.27 | 0.0083054 | 74.6500216 | 0.0000003 |
| 71.54 | 70.92 | 0.0086665 | 71.5402524 | 0.0000035 |
| 67.61 | 66.82 | 0.0116847 | 67.6099628 | 0.0000006 |
| 69.22 | 68.86 | 0.0052008 | 68.7436008 | 0.0068824 |
| 68.87 | 68.86 | 0.0001452 | 68.7436008 | 0.0018353 |
| 65.16 | 65.43 | 0.0041436 | 65.1602436 | 0.0000037 |
| 79.34 | 78.9 | 0.0055458 | 79.1997936 | 0.0017672 |
| 67.46 | 68.86 | 0.0207530 | 68.7436008 | 0.0190276 |
| 74.01 | 74.8 | 0.0106742 | 74.0099721 | 0.0000004 |
| 69.44 | 69.88 | 0.0063364 | 69.4400390 | 0.0000006 |
| 69.81 | 70.16 | 0.0050136 | 69.8099190 | 0.0000012 |
| 67.93 | 67.76 | 0.0025026 | 67.9301176 | 0.0000017 |
| 68.21 | 68.86 | 0.0095294 | 68.7436008 | 0.0078229 |
| 68.63 | 68.8 | 0.0024771 | 68.6491923 | 0.0002796 |
| 70.56 | 68.86 | 0.0240930 | 68.7436008 | 0.0257426 |
| 82.86 | 82.5 | 0.0043447 | 83.1355649 | 0.0033257 |
| **%AAD** |  | 0.63 |  | 0.32 |

***Table S1α2: Calculated values for the percentage prediction error (%PPE) for % antidiabetic activity.***

| **PPE_ALPHA** |  |  |  |  |
| --- | --- | --- | --- | --- |
| **EXPERIMENTAL** | **RSM_OPTIMIZED** | **PPE_RSM** | **ANN_OPTIMIZED** | **PPE_ANN** |
| 68.27 | 68.80 | 0.78 | 68.65 | 0.56 |

***Table S1α3: Calculations of the Root mean square velocity (RMSE) for RSM % antidiabetic activity.***

| **ALPHA_RMSE_RSM_UAE** |  |  |  |
| --- | --- | --- | --- |
| **EXPERIMENTAL** | **RSM_PREDICTED** | **Errors_RSM** | **Square** |
| *69.01* | *68.74* | 0.270 | 0.073 |
| 74.65 | 75.27 | -0.620 | 0.384 |
| 71.54 | 70.92 | 0.620 | 0.384 |
| *67.61* | *66.82* | 0.790 | 0.624 |
| 69.22 | 68.86 | 0.360 | 0.130 |
| *68.87* | *68.86* | 0.010 | 0.000 |
| 65.16 | 65.43 | -0.270 | 0.073 |
| *79.34* | *78.9* | 0.440 | 0.194 |
| 67.46 | 68.86 | -1.400 | 1.960 |
| 74.01 | 74.8 | -0.790 | 0.624 |
| 69.44 | 69.88 | -0.440 | 0.194 |
| 69.81 | 70.16 | -0.350 | 0.122 |
| 67.93 | 67.76 | 0.170 | 0.029 |
| 68.21 | 68.86 | -0.650 | 0.423 |
| *68.63* | *68.8* | -0.170 | 0.029 |
| 70.56 | 68.86 | 1.700 | 2.890 |
| 82.86 | 82.5 | 0.360 | 0.130 |
|  |  | MEAN/Average | 0.486 |
|  |  | RMSE | 0.70 |

***Table S1α4: Calculations of the Root mean square velocity (RMSE) for ANN % antidiabetic activity.***

| **ALPHA_RMSE_ANN_UAE** |  |  |  |
| --- | --- | --- | --- |
| **EXPERIMENTAL** | **ANN_PREDICTED** | **Errors_RSM** | **Square** |
| *69.01* | 69.0825234 | -0.073 | 0.005 |
| 74.65 | 74.6500216 | 0.000 | 0.000 |
| 71.54 | 71.5402524 | 0.000 | 0.000 |
| *67.61* | 67.6099628 | 0.000 | 0.000 |
| 69.22 | 68.7436008 | 0.476 | 0.227 |
| *68.87* | 68.7436008 | 0.126 | 0.016 |
| 65.16 | 65.1602436 | 0.000 | 0.000 |
| *79.34* | 79.1997936 | 0.140 | 0.020 |
| 67.46 | 68.7436008 | -1.284 | 1.648 |
| 74.01 | 74.0099721 | 0.000 | 0.000 |
| 69.44 | 69.4400390 | 0.000 | 0.000 |
| 69.81 | 69.8099190 | 0.000 | 0.000 |
| 67.93 | 67.9301176 | 0.000 | 0.000 |
| 68.21 | 68.7436008 | -0.534 | 0.285 |
| *68.63* | 68.6491923 | -0.019 | 0.000 |
| 70.56 | 68.7436008 | 1.816 | 3.299 |
| 82.86 | 83.1355649 | -0.276 | 0.076 |
|  |  | MEAN/Average | 0.328 |
|  |  | RMSE | 0.57 |

***Graph S1α5: R^2^ values for RSM-predicted % antidiabetic activity.***

***Graph S1α6: R^2^ values for ANN-predicted % antidiabetic activity.***

Time

1

2

3

4

5

6

7

8

9

10

11

12

13

14

15

16

17

18

19

20

1

2

3

4

5

6

7

8

9

10

Yield

TPC

TFC

Antioxidant

Antidiabetic

Temp

S/S ratio

^Independent variables ∈ ℝ³^

^Hidden Layer ∈ ℝ²⁰^

^Hidden Layer ∈ ℝ¹⁰^

^Dependent variables ∈ ℝ¹^


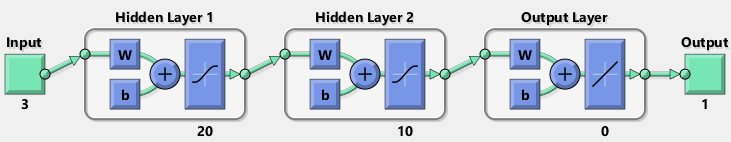


**Figure S2F1:** ANN-model representing independent (input) variables, hidden layers, and dependent (output) layers.


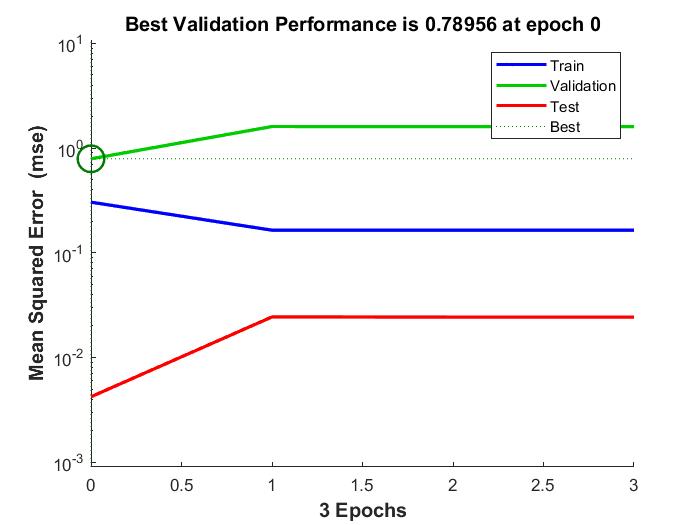

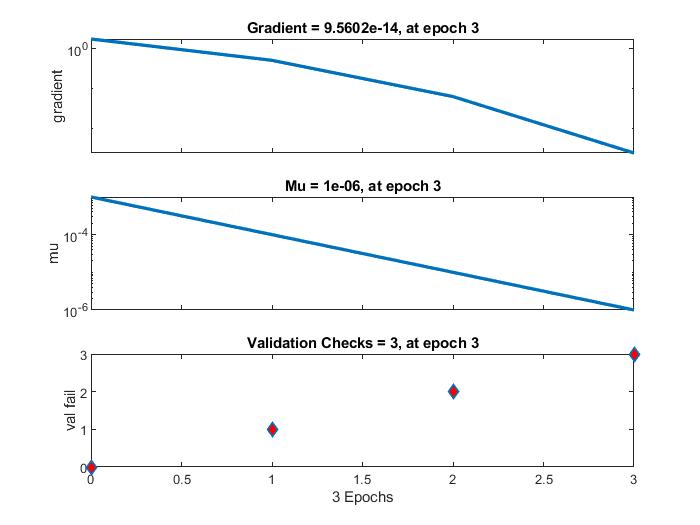

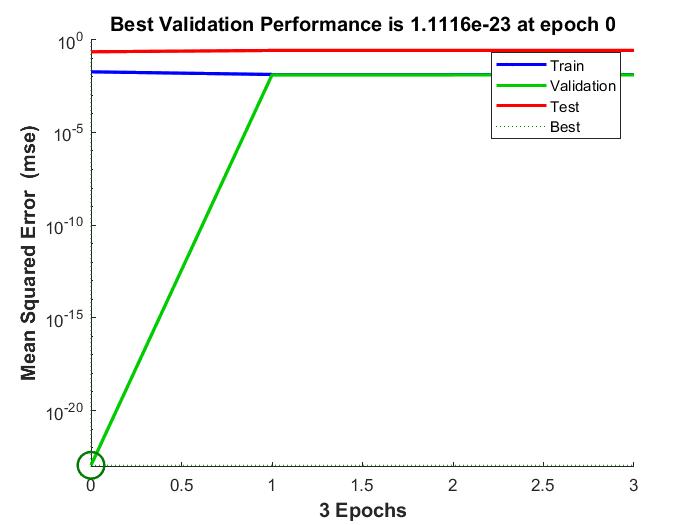

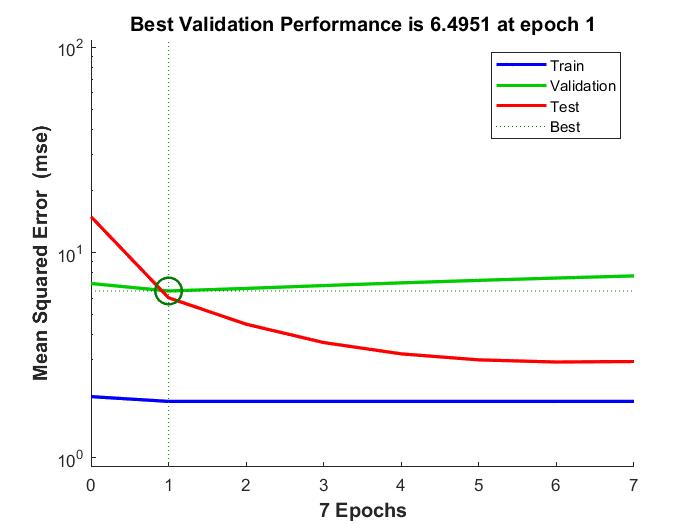

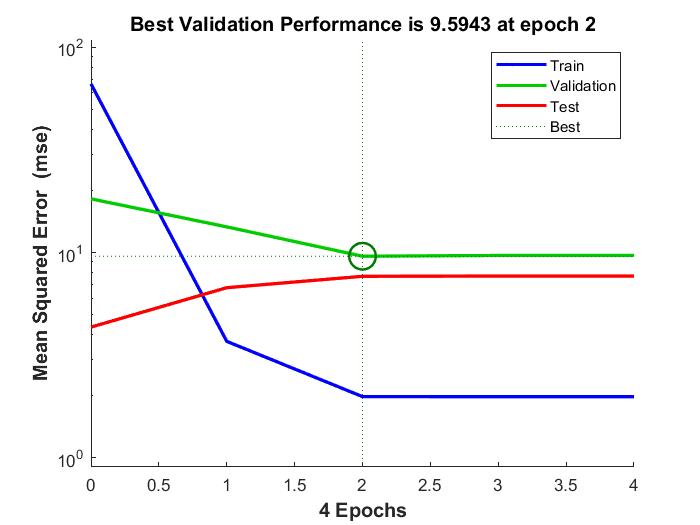

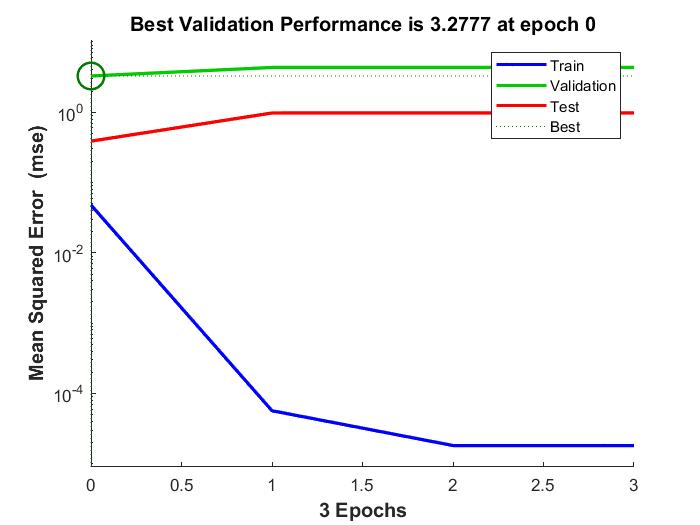


%Yield

TPC

TFC

DPPH

Antidiabetic


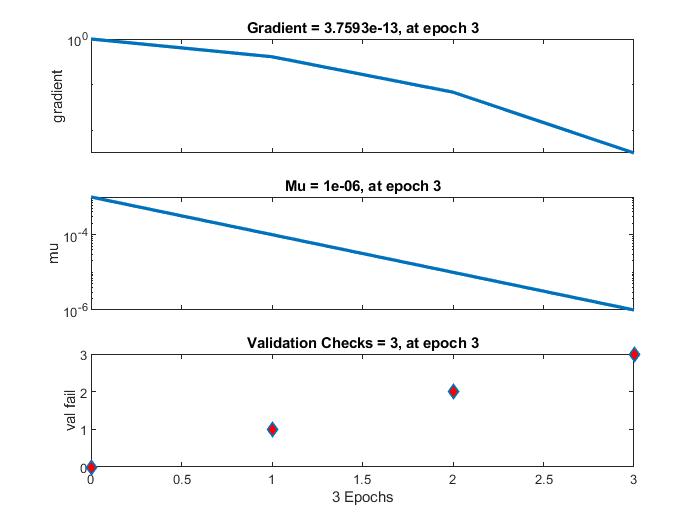


%Yield


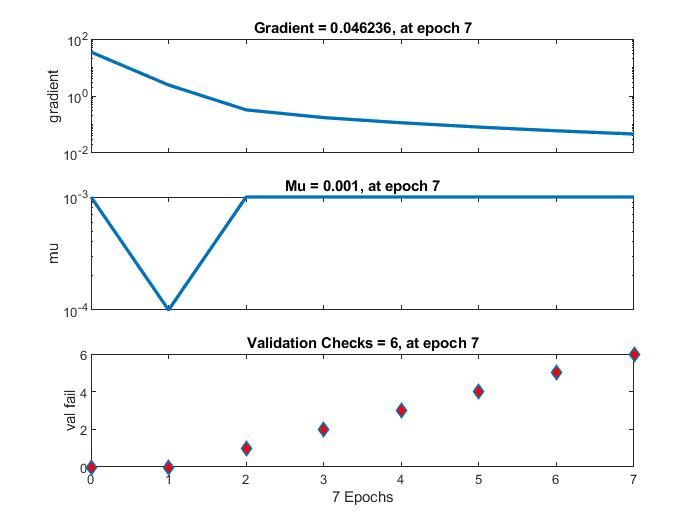


TPC


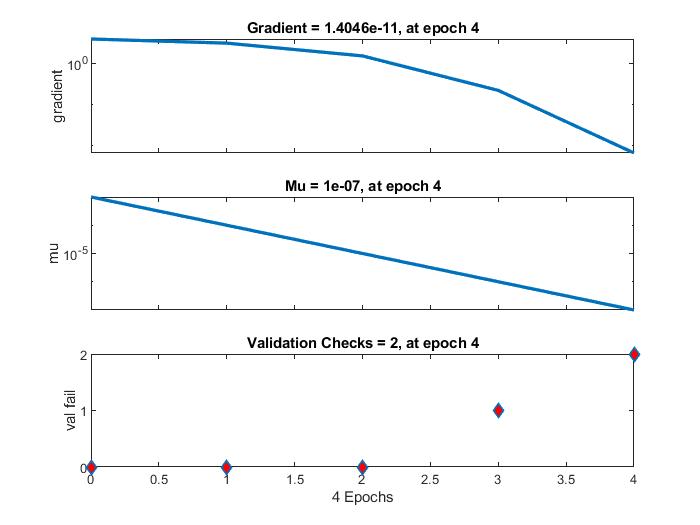


TFC


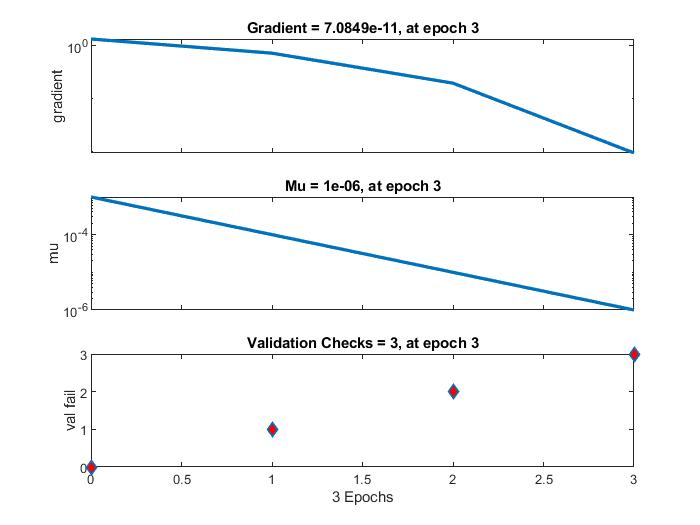


DPPH

Antidiabetic

**s**

**Figure S2F2:** Validation and training state of %yield, TP, TF, antioxidant, and antidiabetic activity generated by ANN.


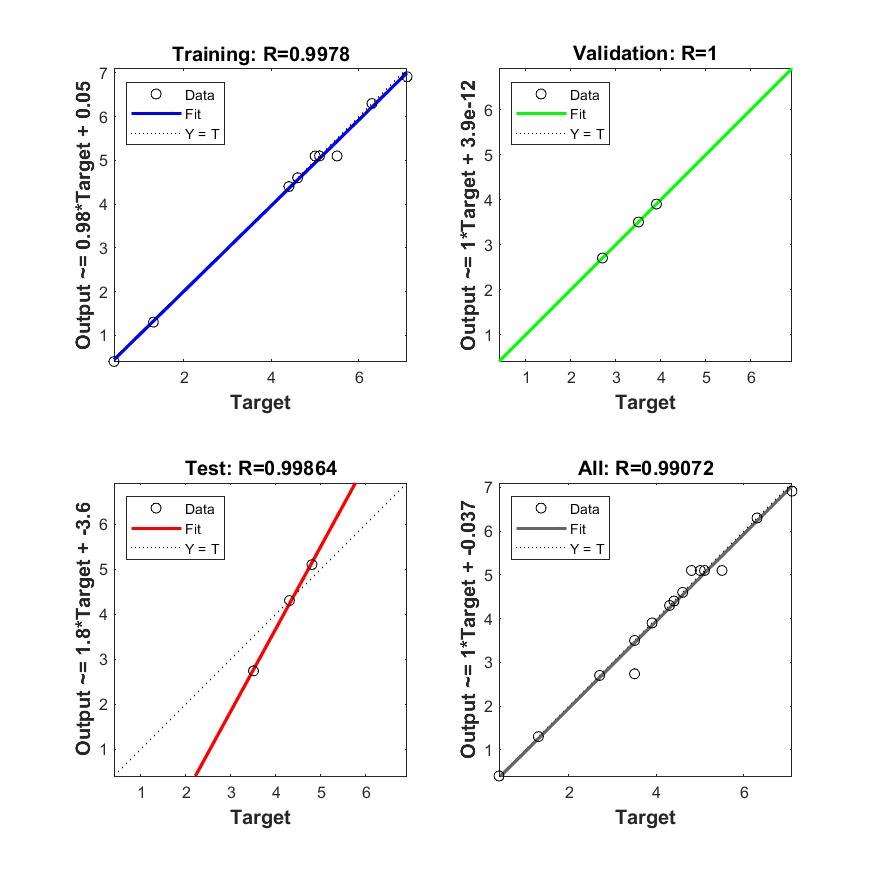


**% Yield**


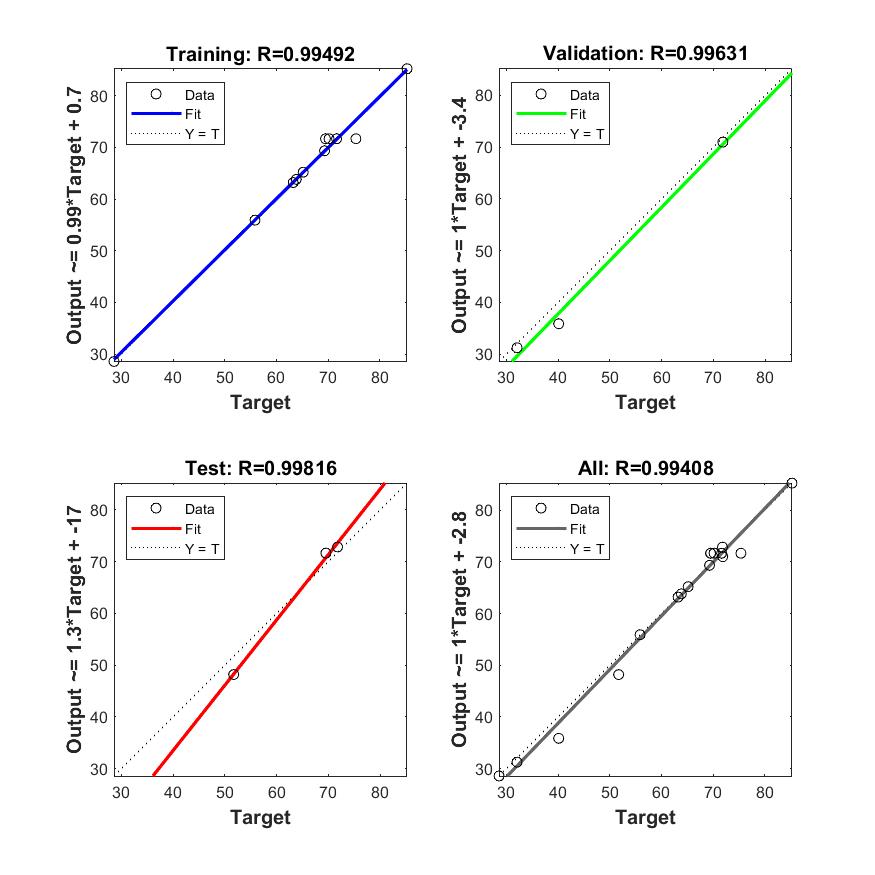


**TPC**


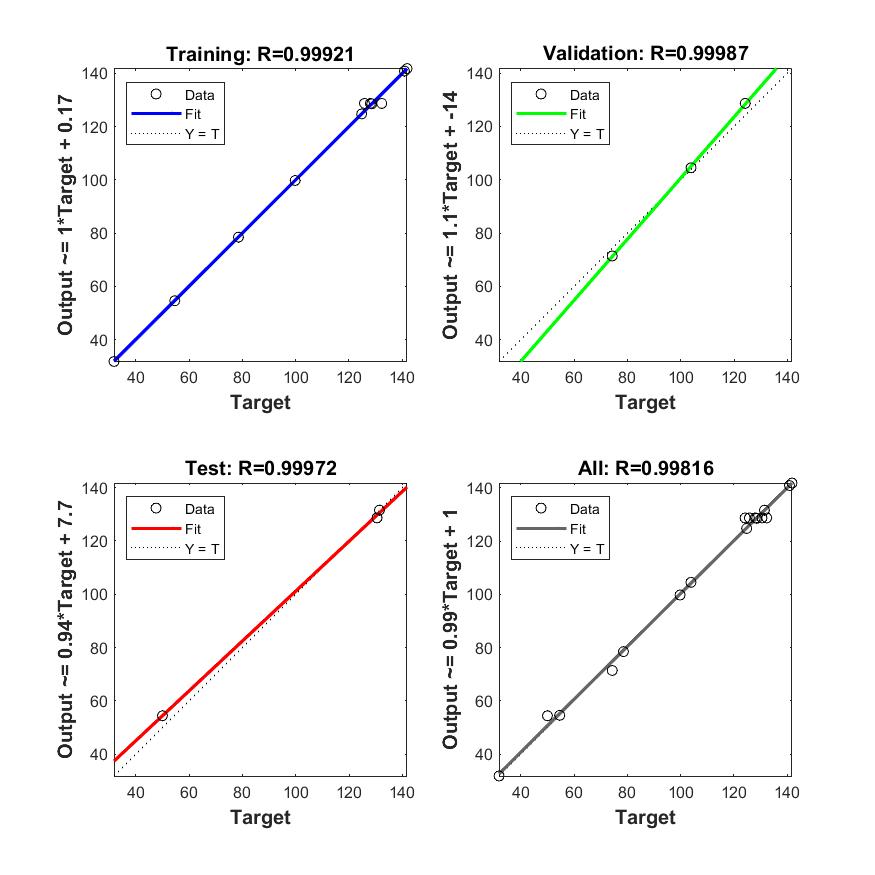


**TFC**


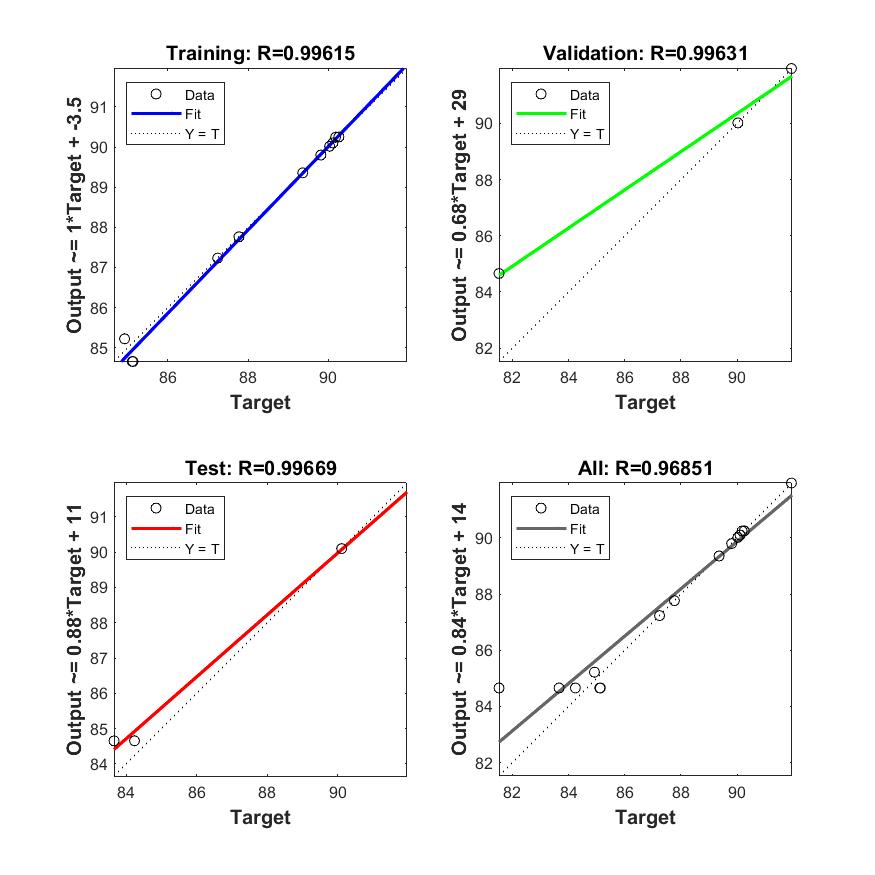


**Antioxidant**


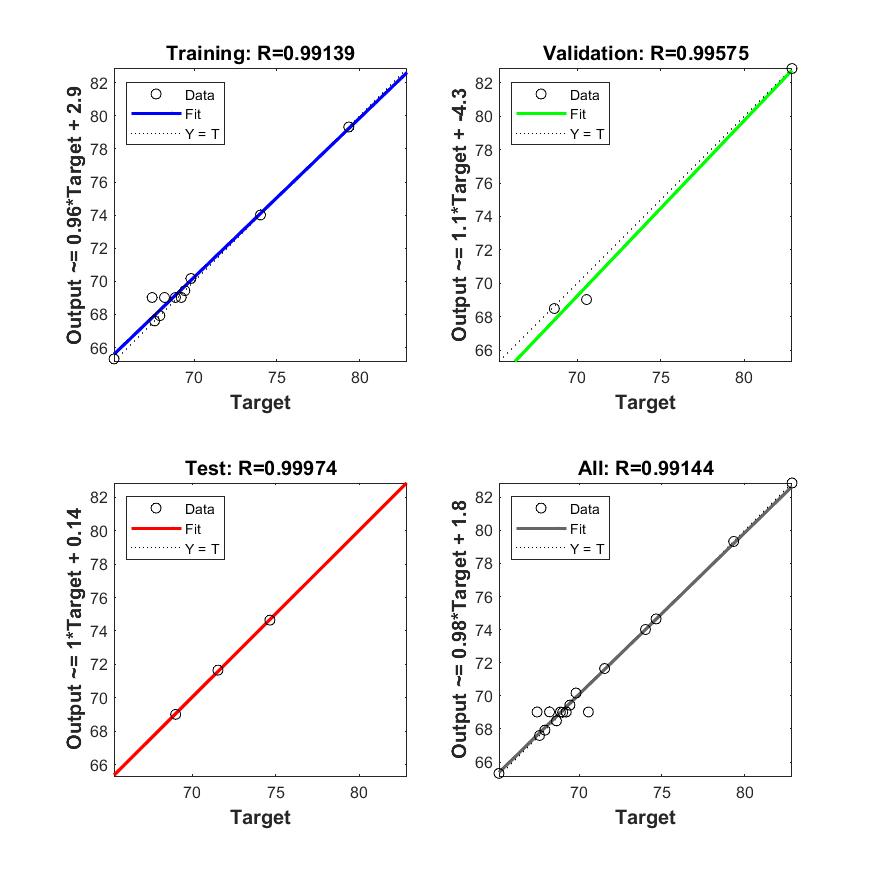


**Antidiabetic**

**Figure S2F3:** Testing, training, validation, and their combined effects (All R) for %yield, TP, TF, antioxidant, and antidiabetic potential.
